# lncRNA2919 Suppresses Rabbit Dermal Papilla Cell Proliferation via *trans*-Regulatory Actions

**DOI:** 10.3390/cells11152443

**Published:** 2022-08-06

**Authors:** Bohao Zhao, Jiali Li, Ming Liu, Shuaishuai Hu, Naisu Yang, Shuang Liang, Xiyu Zhang, Yingying Dai, Zhiyuan Bao, Yang Chen, Xinsheng Wu

**Affiliations:** 1 College of Animal Science and Technology, Yangzhou University, Yangzhou 225009, China; 2 Joint International Research Laboratory of Agriculture & Agri-Product Safety, Yangzhou University, Yangzhou 225009, China

**Keywords:** lncRNA2919, hair follicle growth and development, dermal papilla cells, *trans* regulation, KRTAP11-1, STAT1

## Abstract

Hair follicles (HFs) are complex organs that grow cyclically during mammals’ growth and development. Long non-coding RNAs (lncRNAs) cannot be translated into proteins and play crucial roles in many biological processes. In our previous study, candidate lncRNAs associated with HF cyclic regeneration were screened, and we identified that the novel lncRNA, lncRNA2919, was significantly expressed during catagen. Here, we identified that lncRNA2919 has no coding potentiality and is highly expressed in the cell nucleus, and downregulates HF growth and development-related genes, inhibits cell proliferation, and promotes cell apoptosis in rabbit dermal papilla cells. lncRNA2919 recruits STAT1 to form a compound. As a key transcription factor, STAT1 regulates the transcriptional expression of KRTAP11-1. Our study revealed that lncRNA2919 is involved in HF cyclic regeneration through the *trans*-regulatory lncRNA2919–STAT1–KRTAP11-1 axis. This study elucidates the mechanism through which lncRNA2919 regulates HF growth and development and the role of lncRNA2919 as a new therapeutic target in animal wool production and human hair-related disease treatment.

## 1. Introduction

Hair follicles (HFs) are complicated skin appendant organs composed of various cells, including keratinocytes, dermal sheath cells, melanocytes, and dermal papilla cells (DPCs). These cells participate in HF growth and development, differentiation, and regeneration through regulatory networks, as well as endogenous and exogenous factors [1,2,3,4]. HFs are constantly renewed. The HF cycle stages, namely anagen, catagen, and telogen, are regulated by the control of multiple signaling pathways [5,6]. As they have transcripts of more than 200 nucleotides, long non-coding RNAs (lncRNAs) cannot be translated into proteins and play crucial roles in many biological processes by regulating gene and protein expression, chromatin states, and cellular activities [7,8,9]. lncRNA functions are complicated because lncRNA has a complex structure and expression pattern. In *cis* regulation, lncRNA can act on the adjacent mRNA by regulating the regional chromatin state and gene expression. In *trans* regulation, lncRNA may detach from the transcription site to regulate the remote mRNA through transcriptional activation, adjustment and control of the chromosome structure, and regulation of the gene and protein expression [10].

lncRNA participates in biological processes, such as cellular activities, embryonic development, organ morphogenesis, and disease occurrence [11,12]. In HF biology, lncRNA has vital roles in HF growth and development. For example, HOTAIR, RP11-766N7.3, H19, and UCA1 could regulate HF development via the Wnt/β-catenin signaling pathway in different passage DPCs screened through RNA-Seq [13]. PlncRNA-1 regulated the proliferation and differentiation of HF stem cells (HFSCs) through the Wnt/β-catenin signaling pathway [14]. lncRNA5322 regulated the PI3K/AKT expression and phosphorylation and thus induced HFSC proliferation and differentiation [15]. Furthermore, several lncRNAs related to HF growth and development have been identified, such as lncRNA-000133, lnc_000679, lnc_000344, and lnc108635596 [16,17,18,19]. In our previous study, lncRNA associated with different HF cycle stages were screened; lncRNA2919 was significantly expressed during catagen, and the co-expression/*trans*-regulatory relationship between lncRNA2919 and KRTAP11-1 has been identified [20]. With the *trans*-regulatory pattern, lncRNAs participate in bladder cancer by multiple signaling pathways through the lncRNA–TF–mRNA regulatory network [21]. lncRNA MFI2-AS1 regulated the development process in rectal cancer through the lncRNA–TF–mRNA regulatory network [22]. In skeletal muscle atrophy, with its *trans*-regulatory action, lncMAAT regulated miR-29b expression through SOX6 [23]. Keratin-associated proteins are essential for hair shaft formation, and keratin-associated protein 11-1 (KRTAP11-1) plays crucial roles in HF growth and development by regulating the physical properties of the hair shaft [24,25]. Accordingly, the hypothesis of the *trans*-regulatory module between lncRNA2919 and KRTAP11-1 must be verified.

The current study investigated the role of a novel lncRNA, lncRNA2919, in regulating HF growth and development-related genes in DPCs. lncRNA2919 could inhibit cell proliferation, but promote cell apoptosis in DPCs. Then, the *trans*-transcriptional regulatory network of lncRNA2919–STAT1–KRTAP11-1 was investigated, considering that it plays a negative role in HF growth and development.

## 2. Materials and Methods

### 2.1. Animals

All of the animal experimental procedures in the study were approved by the Animal Care and Use Committee of Yangzhou University. All of the Angora rabbits (age: 6 months) were housed under the same controlled conditions (temperature, water, and diet). For anesthetization, the rabbits were treated with Zoteil 50, and the dorsal skin samples (1 cm^2^) were collected, placed immediately in liquid nitrogen, and stored at −70 °C until use in subsequent experiments for RNA and protein extraction. A portion of the skin samples was fixed in 4% formaldehyde, and paraffin sections were stained with hematoxylin–eosin for histological observations. The iodine solution was applied on the wound to prevent bacterial infection.

### 2.2. Cell Culture

The RAB-9 cells (rabbit skin fibroblasts, ATCC^®^ CRL-1414™) were used for the Dual-luciferase assay. The cells were purchased from ATCC and maintained in a Minimum Essential Medium (Gibco^®^, Grand Island, NY, USA) containing 10% fetal bovine serum (Gibco^®^, Grand Island, NY, USA). DPCs procured from our research group, which were separated from the 6-month-old male Angora rabbit dorsal HF, were maintained in mesenchymal stem cell medium (Sciencell^®^, San Diego, CA, USA). Lipofectamine™ 2000 or 3000 (Invitrogen, Waltham, CA, USA) was used for cell transfection, according to the manufacturer’s instructions, after the cells grown in 6-well (at approximately 1.0 × 10^6^ cells/well) or 24-well (at approximately 2.0 × 10^5^ cells/well) plates reached 80% confluence.

### 2.3. Rapid Amplification of cDNA Ends and Bioinformatics Analysis

According to our previous studies, we obtained raw data from lncRNA-seq (PRJNA479733) and annotated the raw sequence data of lncRNA2919 in the rabbit genome. After the primers were designed using the raw sequence, the total RNA (1 µg) obtained from the Angora rabbit dorsal skin was used for the rapid amplification of 5′ or 3′ cDNA ends (RACE) using the SMARTer^®^ RACE 5′/3′ Kit and 3′ Full RACE Core Set with PrimeScript™ RTase (Takara, Dalian, China), according to the manufacturer’s instructions. The total length sequence of lncRNA2919 was obtained from 5′/3′ RACE, and the sequence information and chromosomal location of the sequence determined were blast to the NCBI, Ensembl, and UCSC databases. The code capacity of lncRNA2919 was predicted through bioinformatics, and the potential open reading frame (ORF) was predicted using ORFfinder (https://www.ncbi.nlm.nih.gov/orffinder/ (accessed on 27 July 2020)) [26]. The potential code capacity was estimated using Coding Potential Calculator 2 (CPC2, http://cpc2.cbi.pku.edu.cn (accessed on 27 July 2020)) [27]. The pcDNA3.1-3 × Flag recombinant plasmids were constructed with ATG insertion in the 5′ end and T, TT insertion in the 3′ end. The primers used are listed in Tables S1 and S2.

### 2.4. Plasmid Preparation, Small Interfering RNA, and Short Hairpin RNA

For constructing the overexpression vector, the total RNA was isolated from the rabbit skin using the RNAsimple total RNA Kit (Tiangen, Beijing, China), according to the manufacturer’s instructions. High-quality rabbit skin cDNA was obtained using the PrimeScript™ 1st Strand cDNA Synthesis Kit (Takara, Dalian, China), and 50 ng cDNA was used for PCR using, which was performed using Phanta Max Super-Fidelity DNA Polymerase (Vazyme, Nanjing, China). The PCR products were purified using MiniBEST Agarose Gel DNA Extraction Kit Ver.4.0 (TakaRa, Dalian, China). The PCR products were cloned into the pcDNA3.1(+) vector (Invitrogen, USA) and transformed into *E. coli* DH5α Competent Cells (TaKaRa, Dalian, China). Then, the plasmids were collected using the EndoFree Maxi Plasmid Kit (Tiangen, Dalian, China). Following the steps, pcDNA3.1-lncRNA2919 was constructed using the full-length sequence of lncRNA2919 obtained through RACE. The overexpression vectors of pcDNA3.1-STAT1 and pcDNA3.1-KRTAP11-1 were constructed. The short hairpin RNA (shRNA)-lncRN2919 and small interfering RNAs (siRNAs) (siRNA-STAT1 and siRNA-KRTAP11-1) were designed and purchased from Shanghai GenePharma Co., Ltd. (Shanghai, China). The aforementioned primers are listed in Table S3.

### 2.5. RNA Isolation and Quantitative RT-PCR Analysis

Nuclear and cytoplasmic RNA extraction was performed using the PARIS™ Kit (Invitrogen), and the total RNA was isolated from the cells and skin using the RNAsimple total RNA Kit (Tiangen, Beijing, China), according to the manufacturer’s instructions. For mRNA verification, total RNA (1 μg) was reverse transcribed using the HiScript II Q Select RT SuperMix for qRT-PCR (Vazyme, Nanjing, China), and cDNA (10 ng) was used for the quantitative mRNA analysis, which was performed using AceQ qPCR SYBR^®^ Green Master Mix (Vazyme, Nanjing, China). For lncRNA verification, the lnRcute lncRNA First-Strand cDNA Kit (Tiangen, Beijing, China) was used for cDNA synthesis, and the lnRcute lncRNA qPCR Kit (SYBR Green, Tiangen, Beijing, China) was used for qRT-PCR. The data were analyzed using QuantStudio^®^ 5 (Applied Biosystems, Massachusetts, USA), according to the manufacturer’s instructions. The relative expression levels were calculated using the 2^−ΔΔCt^ method [28] and glyceraldehyde 3-phosphate dehydrogenase (GAPDH) as the reference gene. U6 RNA served as a positive control for the nuclear gene expression. The specific primers are listed in Table S4.

### 2.6. Western Blotting System (Protein Simple Wes)

The skin and cell samples were collected for detecting the protein expression level. Protein lysates were obtained using the RIPA lysis buffer (PPLYGEN, Beijing, China). The protein concentration was measured using the Enhanced BCA Protein Kit (Beyotime, Shanghai, China). The protein samples were diluted to 0.5 μg/μL for detecting the protein level. The protein (1.5 ng) was analyzed using the automated Western blotting system (Protein Simple Wes) [29], according to the manufacturer’s instructions. The following antibodies were used: 1:2000 anti-GAPDH mouse monoclonal antibody (Abcam), anti-WNT2 rabbit polyclonal antibody (Bioss, Beijing, China), and 1:100 anti-CCND1 mouse monoclonal antibody, anti-LEF1 rabbit polyclonal antibody, anti-KRTAP11-1 mouse polyclonal antibody, and anti-STAT1 mouse monoclonal antibody (Proteintech, Wuhan, China).

### 2.7. Cell Proliferation and Apoptosis Assays

Cell Counting Kit-8 (CCK8, Vazyme, Nanjing, China) was used for the cell proliferation analysis, according the manufacturer’s instructions. The cells were seeded in 96-well plates (1 × 10^4^ cells/wells), and the optical density at 450 nm was determined at 0, 24, 48, and 72 h by using a multimode plate reader (Infinite™ M200 PRO, Tecan, Grödig, Austria). Then, the cells were seeded into 24-well plates (2.0 × 10^5^ cells/wells), and cell apoptosis rates were measured using the Annexin V-FITC Apoptosis Detection Kit (Vazyme, Nanjing, China), according the manufacturer’s instructions. Fluorescence-activated cell sorting was performed using the FACSAria SORP flow cytometer (Becton Dickinson). The results were analyzed using FlowJo V10 software (FlowJo LLC, Ashland, OR, USA).

### 2.8. Dual-Luciferase Assay Verification of the Promoter Region

The promoter region segments of KRTAP11-1 were cloned into pGL3-Basic vectors. The JASPAR database (http://jaspar.genereg.net/ (accessed on 5 October 2020)) was used for predicting TF in the promoter region. The TF binding sites were mutated using the Mut Express II Fast Mutagenesis Kit V2 (Vazyme, Nanjing, China); the primers are listed in Table S5. After cell transfection in 24-well plates (2.0 × 10^5^ cells/wells), the luciferase activity was measured using the dual-luciferase reporter system (Promega, Madison, WI, USA), and the firefly luciferase activity was normalized to the corresponding Renilla luciferase activity.

### 2.9. Electrophoretic Mobility Shift Assay

The nuclear extracts were prepared from DPCs using Nuclear and Cytoplasmic Extraction Reagents (Viagene Biotech, Changzhou, China), and the electrophoretic mobility shift assay (EMSA) was performed using a biotin-labeled EMSA kit (Viagene Biotech, Changzhou, China), according to the manufacturer’s instructions. The sequences of the wild-type and mutant probe are listed in Table S6. Briefly, 1.5 μL of 10× binding buffer, 1.0 μL Poly(dI:dC), 2 μL nuclear proteins (1 μg/μL), and 0.5 µL biotin-labeled probes were incubated together for 20 min at room temperature. For the competition experiments, 1.5 μL of 10× binding buffer, 1.0 μL Poly(dI:dC), 2 μL nuclear proteins, and an unlabeled cold-competitor oligonucleotide or mutant-type oligonucleotide were incubated together for 20 min at room temperature, and then the biotin-labeled probe was added for 20 min at room temperature. The reaction mixtures were separated through 5.5% nondenaturing polyacrylamide gel electrophoresis and transferred to a nylon membrane. Finally, the membrane was incubated with a chemiluminescence substrate buffer and the bands were visualized using CoolImger III (Viagene Biotech, Changzhou, China).

### 2.10. RNA Pull-Down and RNA-Binding Protein Immunoprecipitation Assays

The pull-down assay was performed using the Pierce Magnetic RNA-Protein Pull-Down Kit (Thermo Fisher Scientific, USA), according to the manufacturer’s protocol. The full-length sequence of lncRNA2919 was used for the design of PCR using sense and antisense primers (Table S7). Sense and antisense RNAs were in vitro transcribed using Ribomax Large-Scale RNA Production Systems (Promega, Madison, WI, USA). Then, RNA was labeled with biotin using Biotin RNA Labeling Mix (Roche, Basel, Switzerland), and 50 pmol biotinylated RNA was used for the subsequent operation. Protein extracts (>200 μg) from DPCs were mixed with biotinylated transcripts at 4 °C for 1 h. Streptavidin agarose magnetic beads were incubated with the cell protein lysate to precipitate the RNA–protein complex. This complex was eluted and resolved through sodium dodecyl sulfate-polyacrylamide gel electrophoresis (SDS-PAGE). Then, the gel was subjected to silver staining, and the bands were excised and analyzed through mass spectrometry. The binding proteins were obtained through mass spectrometry, and gene ontology (GO) enrichment was used for the biological analysis of the proteins.

After collecting the DPCs, the RNA-binding protein immunoprecipitation (RIP) assay was performed using Magna RIPTM Kit (Millipore, Nantong, China), following the manufacturer’s guidance. Mouse STAT1 monoclonal antibody (Proteintech, Wuhan, China) and normal mouse immunoglobulin G (IgG; Millipore) were used. The immunoprecipitated RNA and input total RNA were isolated, and qRT-PCR was performed to verify the interaction between lncRNA2919 and STAT1. The primers are listed in Table S8.

### 2.11. Statistical Analysis

The gene or lncRNA relative expression, luciferase activity, OD value, and apoptosis rates were analyzed using two-tailed Student’s *t*-test or ANOVA with SPSS 25.0 (SPSS Inc., Chicago, IL, USA). For each analysis, a minimum of three biological replicates was used. All of the error bars in the results represent the mean ± SEM.

## 3. Results

### 3.1. Identification and Characterization of lncRNA2919

**Figure 1:**
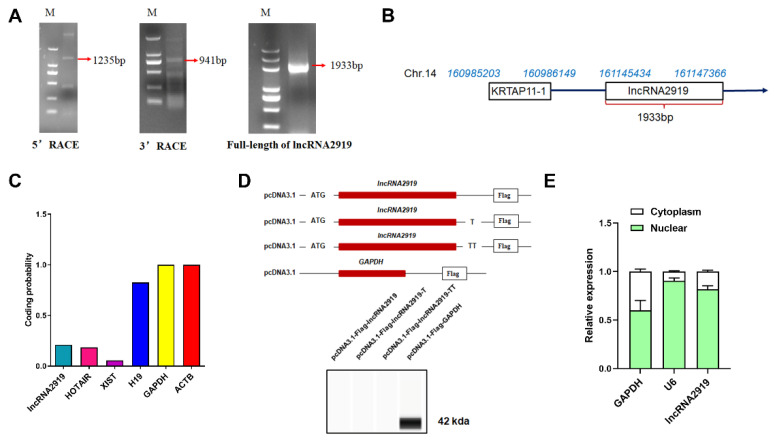
Cloning and analysis of the lncRNA2919 full-length sequence. (**A**) The lncRNA2919 full-length sequence was obtained through RACE. M indicated the DL 2000 DNA marker. (**B**) The information of lncRNA2919 located in the rabbit genome. The arrow indicates the direction of transcription (5′ to 3′). (**C**) The coding probability prediction of lncRNA2919 through CPC2. (**D**) The coding ability of lncRNA2919 was verified through the detection of the pcDNA3.1-Flag vector. (**E**) lncRNA2919 expression in the nucleus and cytoplasm of DPCs.

lncRNA2919 is located between *KRTAP11-1* and *TIAM1* on rabbit chromosome 14. Through a function analysis between lncRNA and mRNA, the co-location/cis relationship between lncRNA2919 and the gene (TIAM1), as well as the co-expression/*trans*-regulatory relationship between lncRNA2919 and seven genes, such as *KRTAP11-1*, *TTC7B*, *TRIT1*, and *TM4SF1*, were identified (Table S9). Furthermore, 5′ RACE and 3′ RACE demonstrated that lncRNA2919 was 1933-bp long (Figure 1A) and was located on chr.14:161145434–161147366 following the direction of transcription (5′ to 3′) (Figure 1B). The prediction of the ORFfinder showed that lncRNA2919 contained 14 ORFs, with the longest ORF being 285 nt. The longest ORF could encode 94 amino acids, less than the number of amino acids coded by a coding gene. CPC2 showed that the coding capacity of lncRNA2919 was 0.2104 (Table S10), similar to that of HOTAIR (Figure 1C). pcDNA3.1-Flag and Western blot assays revealed that lncRNA2919 had no protein-coding potentiality (Figure 1D). The cell fractionation assays, followed by qRT-PCR, showed that approximately 78% of the lncRNA2919 transcript resides in the nucleus of DPCs (Figure 1E).

### 3.2. lncRNA2919 Plays a Negative Role in DPC Proliferation

**Figure 2:**
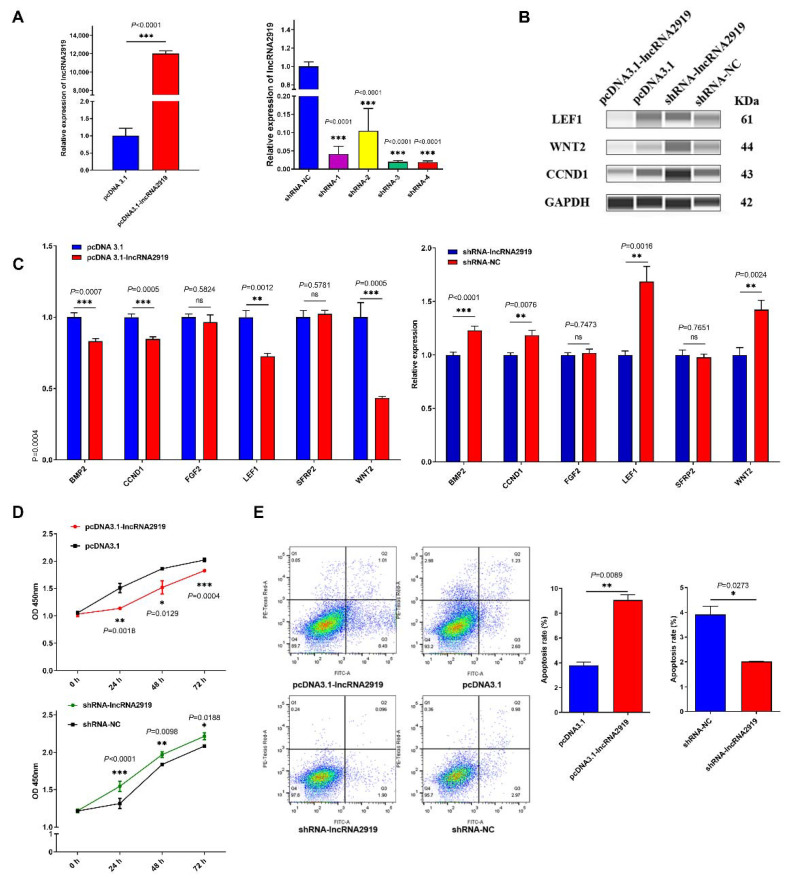
Negative role of lncRNA2919 in HF cycling and growth. (**A**) lncRNA2919 expression was detected after lncRNA2919 overexpression and knockdown in DPCs. (**B**) lncRNA2919 regulated the expression of HF cycling and growth-related proteins after lncRNA2919 overexpression and knockdown in DPCs. (**C**) lncRNA2919 regulated the mRNA expression of HF cycling and growth-related genes after lncRNA2919 overexpression and knockdown in DPCs. (**D**) lncRNA2919 inhibited the proliferation of DPCs. (**E**) lncRNA2919 promoted the apoptosis of DPCs. Data are presented as mean ± SEM, ns indicates not significant. A two-tailed paired *t*-test was used for data analyses.

To determine the role of lncRNA2919 in HF cyclic regeneration, the pcDNA3.1-lncRNA2919 vector was constructed and transfected into DPCs. The qRT-PCR result showed that pcDNA3.1-lncRNA2919 could upregulate the lncRNA2919 expression. lncRNA2919 knockdown in DPCs was performed using lentivirus-mediated shRNAs. shRNA-4 achieved a more effective knockdown efficiency (Figure 2A). lncRNA2919 overexpression could decreased LEF1, CCND1, and WNT2 protein expression (Figure 2B). HF growth- and development-related genes were detected. The results showed that lncRNA2919 could suppress *BMP2*, *CCND1*, *LEF1*, and *WNT2* mRNA expression (Figure 2C). Furthermore, we examined DPC proliferation and apoptosis after lncRNA2919 overexpression and knockdown. The results showed that lncRNA2919 could significantly inhibit DPC proliferation (Figure 2D) and promote their apoptosis (Figure 2C).

### 3.3. lncRNA2919 Recruits STAT1 Protein

**Figure 3:**
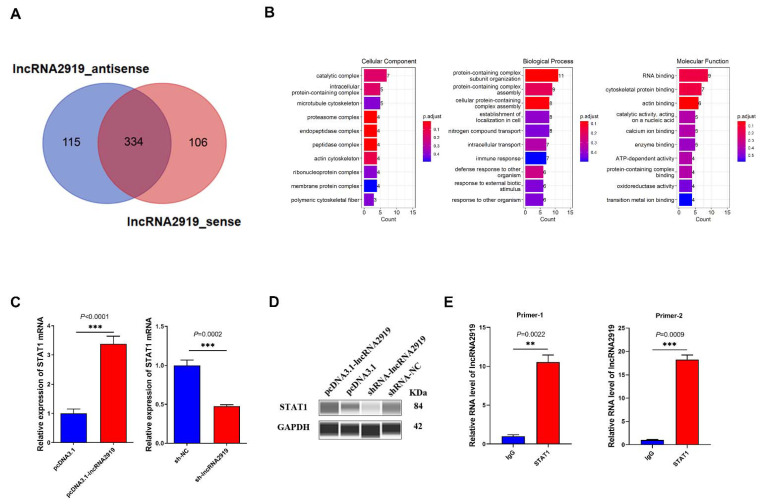
lncRNA2919 regulated the binding protein STAT1. (**A**) Venn diagram shows the number of proteins obtained from the silver staining band of lncRNA2919 from in vitro transcription through mass spectrometry after the RNA pull-down assay. (**B**) The biological function of binding proteins for lncRNA2919 were analyzed through GO term enrichment. (**C**) The STAT1 mRNA expression level after lncRNA2919 overexpression and knockdown. (**D**) The STAT1 protein expression level after lncRNA2919 overexpression and knockdown. (**E**) The interaction between STAT1 and lncRNA2919 was verified through the RIP-qPCR assay. The enrichment of STAT1 and lncRNA2919 was measured through qPCR and normalized to input. The data are presented as mean ± SEM. A two-tailed paired *t*-test was used for data analyses.

To further investigate the downstream molecular mechanism of lncRNA, we identified the interacting proteins of lncRNA2919 using RNA pull-down and mass spectrometry analysis. In the in vitro transcript, lncRNA2919 transcription obtained approximately 1-kb RNA sequence (Figure S1A); biotinylated antisense RNA of lncRNA2919 was used as controls. The lncRNA–protein complex was observed through SDS-PAGE (Figure S1B). In total, 555 proteins were identified that may bind to lncRNA2919_sense and lncRNA2919_antisense, and 106 interacting proteins may specifically bind to lncRNA2919_sense, which excluded the interacting proteins of lncRNA2919_antisense, and 115 and 106 interacting proteins of lncRNA2919_antisense and lncRNA2919_sense, respectively, were found after the mass spectrum identification of proteins (Figure 3A, Table S11). In addition, 15 proteins were identified in the differential band between lncRNA2919_sense and lncRNA2919_antisense (Table S11). Moreover, GO enrichment analysis was performed to reveal the biological function of the lncRNA2919-interacting proteins. Most GO terms were identified for the protein-containing complex subunit organization, RNA binding, protein-containing complex cytoskeletal assembly, and protein binding (Figure 3B). Then, we identified the skin and HF development-related proteins, for example STAT1, KRT16, and ADAR, which may relate to the GO terms, such as hair cycle, morphogenesis of an epithelium, and keratinization. The GO enrichment analysis of the differential band between lncRNA2919_sense and lncRNA2919_antisense showed that proteins may enrich in the keratin family (KRT2, KRT5, and KRT10), which participated in keratinocyte development, proliferation, migration, and activation.

As a key TF, STAT1 plays crucial roles in cell proliferation and differentiation, cell apoptosis, and angiogenesis. We noticed that lncRNA2919 may recruit STAT1, and we verified the lncRNA2919–STAT1 relationship. In DPCs, lncRNA2919 overexpression may upregulate STAT1 mRNA and protein expression, and lncRNA2919 knockdown could downregulate STAT1 mRNA and protein expression (Figure 3C,D). Meanwhile, endogenous STAT1 protein was immunoprecipitated using STAT1 antibody, and the results of RIP-qPCR showed the significant lncRNA2919 expression in the immunoprecipitation (Figure 3E), indicating the formation of a lncRNA2919–STAT1 complex in DPCs.

### 3.4. lncRNA2919 Regulates HF Cyclic Regeneration by the trans-Regulatory Axis of lncRNA2919–STAT1–KRTAP11-1

**Figure 4:**
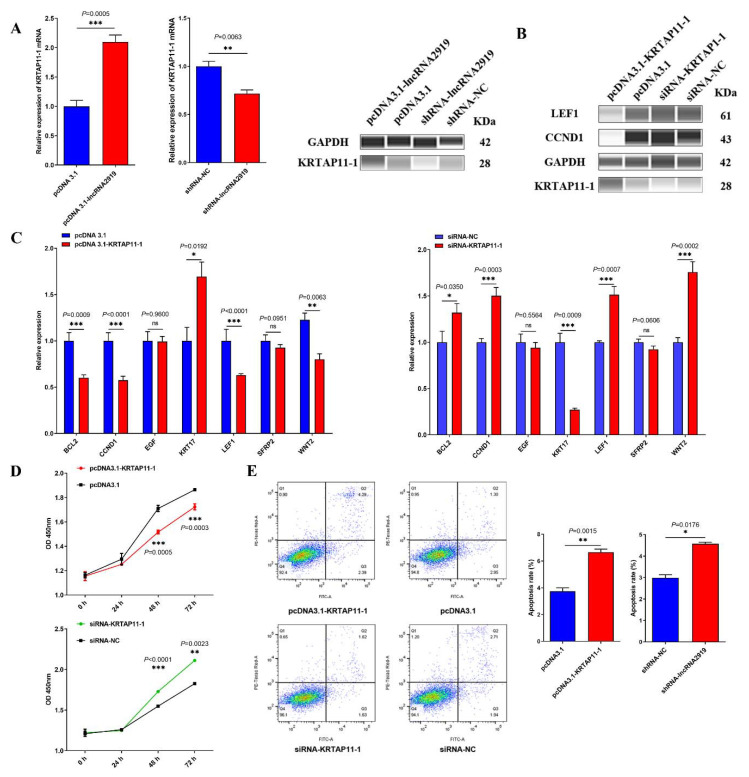
lncRNA2919 regulated the KRTAP11-1 expression. (**A**) lncRNA2919 overexpression and knockdown regulated the KRTAP11-1 mRNA and protein expression levels. (**B**) KRTAP11-1 regulated the expression of HF cycling and growth-related proteins. (**C**) KRTAP11-1 regulated the mRNA expression of HF cycling and growth-related genes after KRTAP11-1 overexpression and knockdown in DPCs. (**D**) Cell proliferations were estimated on the basis of KRTAP11-1 overexpression and knockdown in DPCs. (**E**) Cell apoptosis rates were determined on the basis of KRTAP11-1 overexpression and knockdown in DPCs. Data are presented as mean ± SEM, ns indicates not significant. A two-tailed paired *t*-test was used for the data analyses.

According to our previous study, a *trans*-regulatory relationship existed between lncRNA2919 and KRTAP11-1. lncRNA2919 overexpression significantly upregulated the *KRTAP11-1* mRNA and protein expression, and lncRNA2919 knockdown significantly decreased the KRTAP11-1 mRNA and protein level (Figure 4A). Moreover, the role of KRTAP11-1 in the regulation of HF cyclic regeneration was determined. First, we obtained the full-length mRNA of *KRTAP11-1* through RACE (Figure S2A); the mRNA sequence of KRTAP11-1 was 495 bp. Then, KRTAP11-1 overexpression and knockdown was performed in DPCs (Figure S2B). KRTAP11-1 overexpression could increase the KRTAP11-1 protein expression, but decrease the LEF1 and CCND1 protein expression (Figure 4B). qRT-PCR showed that KRTAP11-1 overexpression increased the *KRT17* mRNA expression and suppressed the *WNT2*, *LEF1*, *CCND1*, and *BCL2* expression. Conversely, KRTAP11-1 knockdown decreased the *KRT17* mRNA expression and increased the *WNT2*, *LEF1*, *CCND1*, and *BCL2* mRNA expression (Figure 4C). To reveal the role of KRTAP11-1 in DPCs, cell apoptosis and proliferation were estimated after KRTAP11-1 overexpression and knockdown. The results showed that KRTAP11-1 inhibited cell proliferation after 48 h, whereas KRTAP11-1 knockdown promoted it after 48 h (Figure 4D). Furthermore, KRTAP11-1 overexpression increased the cell apoptosis rate, whereas KRTAP11-1 knockdown decreased the rate (Figure 4E).

**Figure 5:**
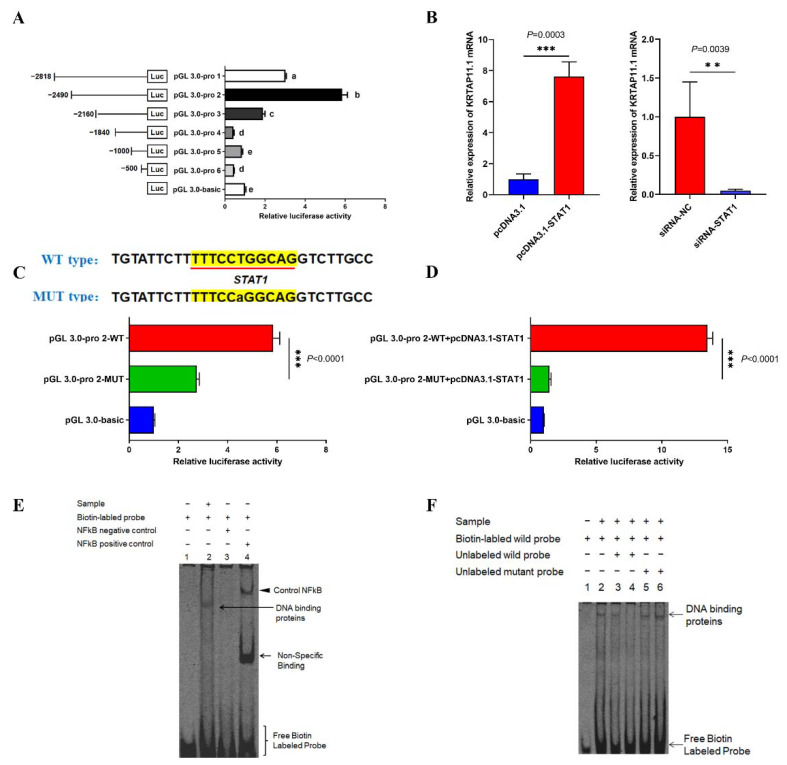
STAT1 regulated KRTAP11-1 transcriptional expression. (**A**) The detection of luciferase activity in the KRTAP11-1 promoter region. (**B**) STAT1 promoted *KRTAP11-1* mRNA expression after STAT1 overexpression and knockdown. (**C**) Luciferase activities were detected after transfection of the STAT1 wild-type vector and mutant vector in the KRTAP11-1 promoter region. (**D**) Luciferase activities were detected after the co-transfection of pcDNA3.1-STAT1 and the STAT1 wild-type vector and mutant vector in the KRTAP11-1 promoter region. (**E**) The binding relationship between STAT1 and the KRTAP11-1 promoter was verified on the basis of the popular response of EMSA. (**F**) The binding relationship between STAT1 and the KRTAP11-1 promoter was verified on the basis of the competitive response of EMSA. The volume of unlabeled oligonucleotides was 33-fold higher than that of the labeled oligonucleotides in the third and fifth lanes. The volume of unlabeled oligonucleotides was 100-fold higher than that of the labeled oligonucleotides in the fourth and sixth lanes. Data are presented as mean ± SEM. A two-tailed paired *t*-test was used for the data analyses. One-way ANOVA with adjusted multiple-comparison was used for the data analyses; different letters indicate the significant differences (*p* < 0.05).

Next, we investigated KRTAP11-1 promoter activities through the luciferase reporter assays. The results revealed the highest luciferase activity at −2490 to −2160 loci in the promoter region, indicating that the core promoter region was located on the −2490 to −2160 loci (Figure 5A). Then, the TFs of the core promoter region were predicted. Interestingly, the TF binding site of the lncRNA2919-interacting protein STAT1 is located in the core promoter region of KRTAP11-1. Then, pcDNA3.1-STAT1 and siRNA-STAT1 were used to determine the *STAT1* mRNA expression level after overexpression and knockdown (Figure S3). STAT1 overexpression increased the *KRTAP11-1* mRNA expression, whereas STAT1 knockdown inhibited the *KRTAP11-1* gene expression (Figure 5B). The site mutation and luciferase reporter assays were used for further verification of the relationship between the TF STAT1 and promoter luciferase activity. The results showed that the site mutant of STAT1 in the KRTAP11-1 promoter region could significantly downregulate the relative luciferase activity (Figure 5C). STAT1 overexpression could significantly promote the relative luciferase activity of the promoter region (Figure 5D). Finally, the binding site of STAT1 in the KRTAP11-1 promoter region was further confirmed through EMSA, indicating that STAT1 could bind to the KRTAP11-1 promoter region (Figure 5E). Unlabeled STAT1 probes competitively disrupted this binding capacity, indicating that STAT1 may bind to the KRTAP11-1 promoter region (Figure 5F).

## 4. Discussion

HF cyclic regeneration is controlled by the interaction of many signaling pathways and factors, which may help in deeply understanding embryonic development, regulation of the metabolism and the endocrine system, blood supply, and nerve conduction in organisms [6,30,31,32,33]. As a type of ncRNA, lncRNAs play pivotal roles in the HF cycle and development. They could regulate cell proliferation, apoptosis, and cycle through their interaction with genes and proteins. They participate in the dynamic process of skin and HF morphogenesis and HF growth and development [14,15,34,35,36]. The lncRNA XLOC_008679 could play a role in anagen of the HF cycle by targeting KRT35 [37], and the lncRNA H19 expressed in anagen regulates wool fiber growth in cashmere goats [35]. LncRNA-PCAT1 regulates the cell proliferation and activity of DPCs through the Wnt/β-catenin signaling pathway by the miR-329/Wnt10b axis [38]. In our study, we identified that lncRNA2919 is highly expressed in the catagen of the HF cycle [20]. The full-length sequence of lncRNA2919 was obtained, and bioinformatics showed the co-location/cis and co-expression/*trans*-regulatory relationship between lncRNA2919 and mRNA. lncRNA2919 may regulate TIAM1 through the co-location/cis relationship. Studies have shown that TIAM1 is expressed in the HF and affects HF formation [39]. KRTAP11-1 plays a crucial role in the assemble of keratin bundles, formation of hair fibers, and hair quality [24,40]. The prediction results showed that lncRNA2919 may regulate KRTAP11-1 by the co-expression/*trans*-regulatory relationship. lncRNA2919 may regulate hair growth and development by the cis/*trans*-regulatory mechanism. The vast majority of lncRNAs do not have the ability to encode proteins, but a few lncRNAs could encode small peptides [41,42]. We identified that lncRNA2919 displayed no protein-coding potentiality, and lncRNA2919 was highly expressed in the cell nucleus, suggesting that lncRNA2919 may play a role in organisms by modifying the chromatin structure and recruiting proteins [43,44,45]. After lncRNA2919 overexpression and knockdown, qRT-PCR showed that lncRNA2919 could significantly downregulate HF growth and development-related genes, such as *BMP2* [46], *CCND1* [47], *LEF1* [48], and *WNT2* [49]. lncRNA2919 could promote cell apoptosis and inhibit the proliferation of DPCs, indicating that lncRNA2919 may regulate HF cyclic regeneration by influencing the cell activities.

lncRNA regulates genes and proteins through the *trans*-regulatory mechanism, including the regulation of the enhancer and promoter, scaffolding of the protein complex, and binding to the protein and RNA [10]. Generally, lncRNA–TF–mRNA is one of the most crucial relationships in the *trans*-regulatory mechanism that plays major roles in the vital movement and disease occurrence. For example, lncRNA BC041488 regulates CDK1 expression through the *trans* regulation of TF SRF [21]. NONMMUT034790.2 mediates TF LEF1 to regulate the SMAD7 via the co-expression/*trans*-regulatory network [50]. We determined the binding proteins of lncRNA2919, such as KRT16 and STAT1, through the RNA pull-down assay. KRT16 is the key molecule affecting the HF structure and HF growth and cycle [51,52]. STAT1 prompted the promoter activities to regulate keratinocyte differentiation in the HF cycle [53]. lncRNA2919 could recruit STAT1 to increase the gene and protein expression. As a key TF, STAT1 may specifically bind to the KRTAP11-1 promoter region. The *trans* regulation of lncRNA2919–STAT1–KRTAP11-1 has been verified, confirming that lncRNA2919 regulates the transcriptional expression of KRTAP11-1. We determined that KRTAP11-1 could regulate KRT17, the key molecule in HF growth and development. KRT17 is expressed in the outer root sheath and hair shaft at the anagen and in the hair sheath at the catagen and telogen [54,55]. KRTAP11-1 downregulates the expression of antiapoptotic genes *CCND1* and *BCL2*, which play key roles in the HF morphology and HF cycle [47,56]. In the study, KRTAP11-1 may promote cell apoptosis and inhibit cell proliferation, indicating KRTAP11-1 is a key player in HF cyclic regeneration. Hence, the regulatory relationship of lncRNA2919–STAT1–KRTAP11-1 plays a negative role in HF cyclic regeneration.

## 5. Conclusions

lncRNA2919 plays a negative role in the regulation of HF growth and development-related genes. lncRNA2919 could promote cell apoptosis and inhibit the proliferation of DPCs. Furthermore, STAT1 is recruited by lncRNA2919 to form a compound, which could regulate the transcriptional expression of KRTAP11-1 through the co-expression/*trans*-regulatory mechanism of lncRNA2919–STAT1–KRTAP11-1. The research will provide a new perspective on how lncRNA regulates HF growth and development and HF cyclic regeneration, thus filling up the research gap in HF biology and hair disease therapy.

## Data Availability

All data supporting our findings are included in the manuscript.

## Supplementary Materials

The following supporting information can be downloaded at: https://www.mdpi.com/article/10.3390/cells11152443/s1. Figure S1: (A) The result of in vitro transcription of lncRNA2919 through RNA electrophoresis. The size of the marker (M) was 5 kb, 3 kb, 2 kb, 1.5 kb, 1 kb, 750 bp, 500 bp, 250 bp, and 100 bp. (B) The result of in vitro transcription of lncRNA2919 determined through silver staining. The size of the marker (M) was 116 KDa, 66.2 KDa, 45 KDa, 35 KDa, 25 KDa, 18.8 KDa, and 14.5 KDa. Figure S2: (A) Full-length mRNA sequence of KRTAP11-1 was obtained through RACE. (B) The KRTAP11-1 mRNA expression level of after KRTAP11-1 overexpression and knockdown in DPCs. Data are presented as mean ± SEM. A two-tailed paired t-test was used for the data analyses. Figure S3: STAT1 mRNA expression level after STAT1 overexpression and knockdown in DPCs. Data are presented as mean ± SEM. A two-tailed paired t-test was used for the data analyses. Table S1: Primers used for RACE. Table S2: Primers used for constructing the pcDNA3.1-Flag vector. Table S3: Primers used for constructing the vector and sequences of shRNA and siRNA. Table S4: Information of primers used for qRT-PCR. Table S5: Primers used for constructing the luciferase reporter vector. Table S6: Probe sequences of the TF in the promoter region for EMSA. Table S7: PCR primers of sense and antisense in vitro transcription. Table S8: RT-PCR primers used in the RIP assay. Table S9: Prediction of the target gene of lncRNA2919. Table S10: Coding probability prediction of lncRNA2919 by CPC2. Table S11: The interacting proteins of lncRNA2919. Supplementary File S1. Origin figure of the Wes analysis. Supplementary File S2. The sequence of lncRNA2919, *KRTAP11-1* mRNA, and *STAT1* CDS in the study.
