# Supplementary material for: lncRNA2919 Suppresses Rabbit Dermal Papilla Cell Proliferation via trans-Regulatory Actions
Source: Cells. 2022 Aug 6;11(15):2443. doi: 10.3390/cells11152443 (PMC9368379; doi:10.3390/cells11152443)
Supplement: Supplementary file 1 [file cells-11-02443-s001.zip › Supplementary file S1.pdf]

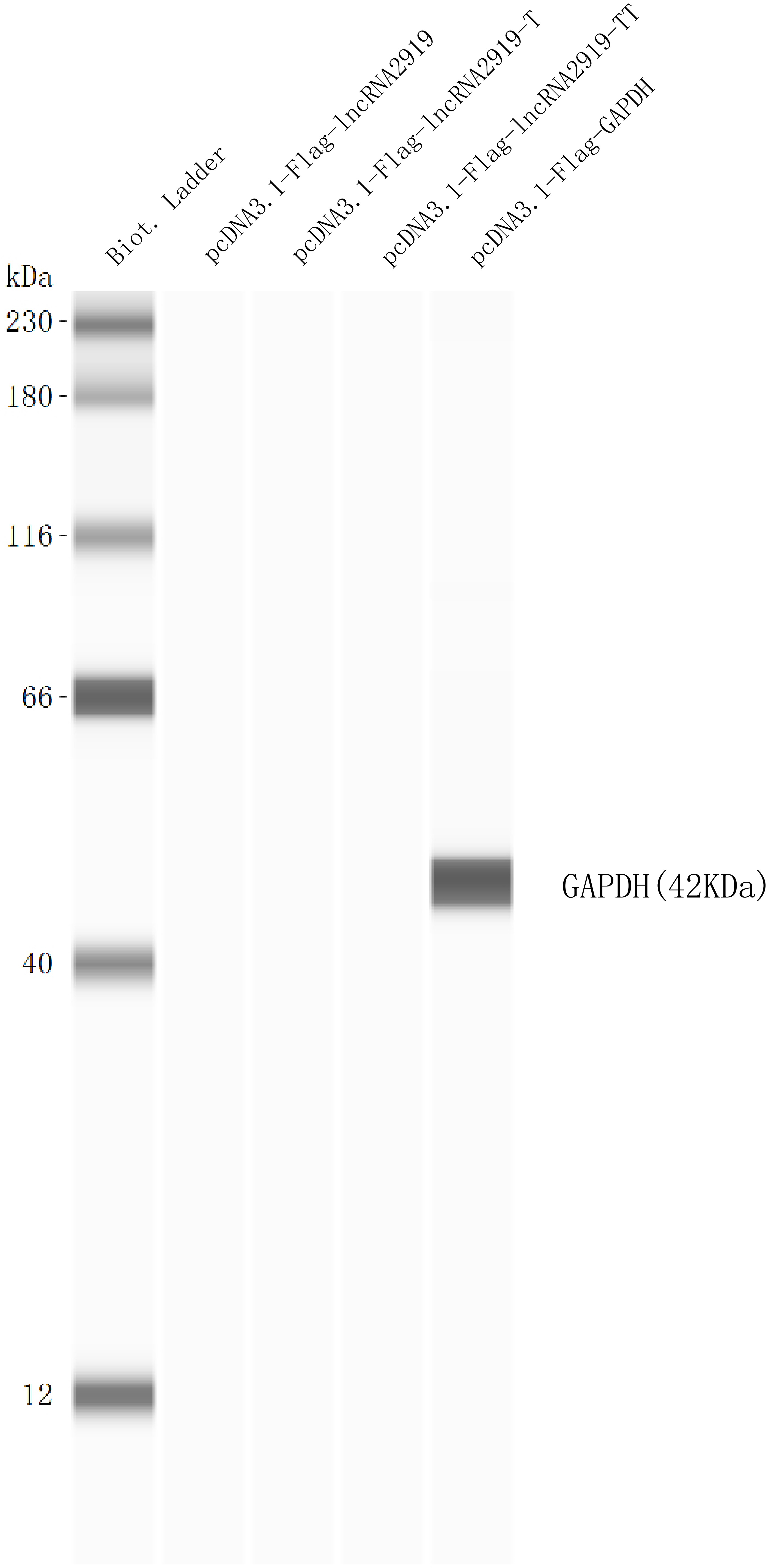
Biot. Ladder
pcDNA3.1-Flag-lncRNA2919
pcDNA3.1-Flag-lncRNA2919-T
pcDNA3.1-Flag-lncRNA2919-TT
pcDNA3.1-Flag-GAPDH
kDa
230
180
116
66
40
12
GAPDH(42KDa)

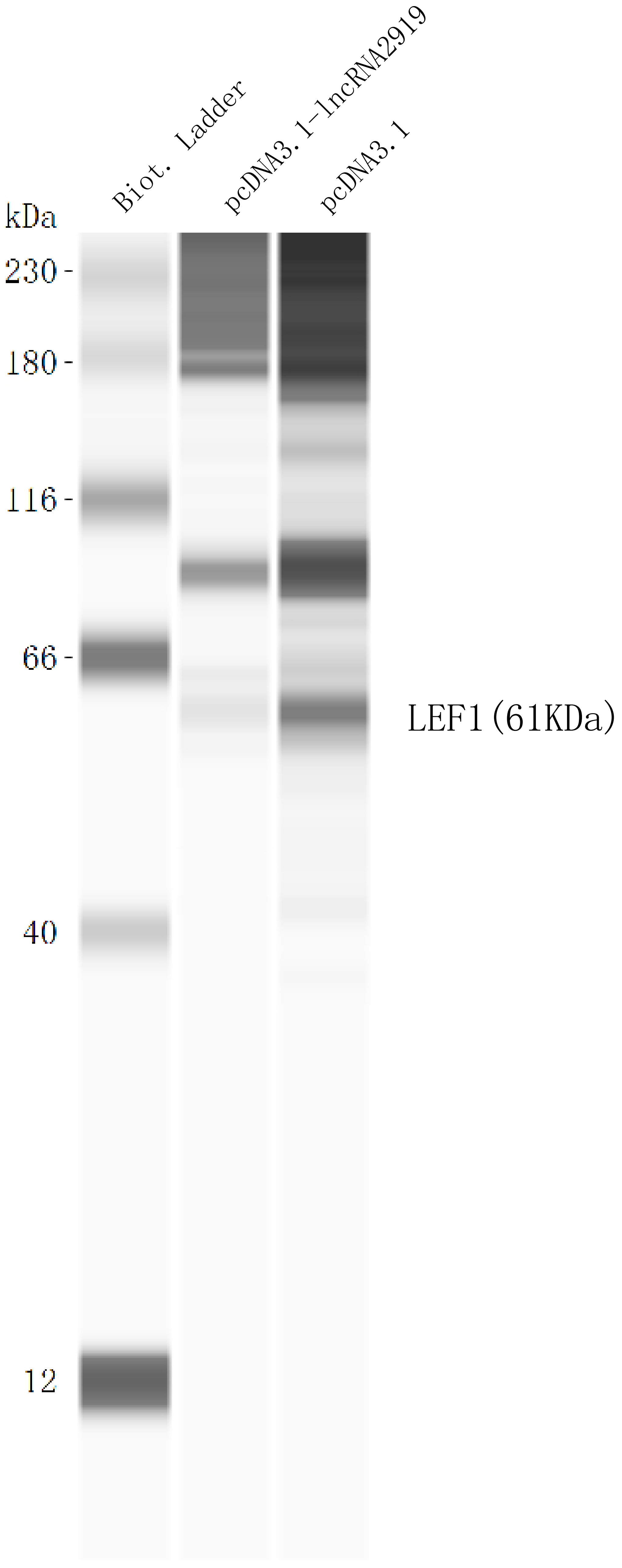
Biot. Ladder
pcDNA3.1-lncRNA2919
pcDNA3.1
kDa
230
180
116
66
40
12
LEF1(61KDa)

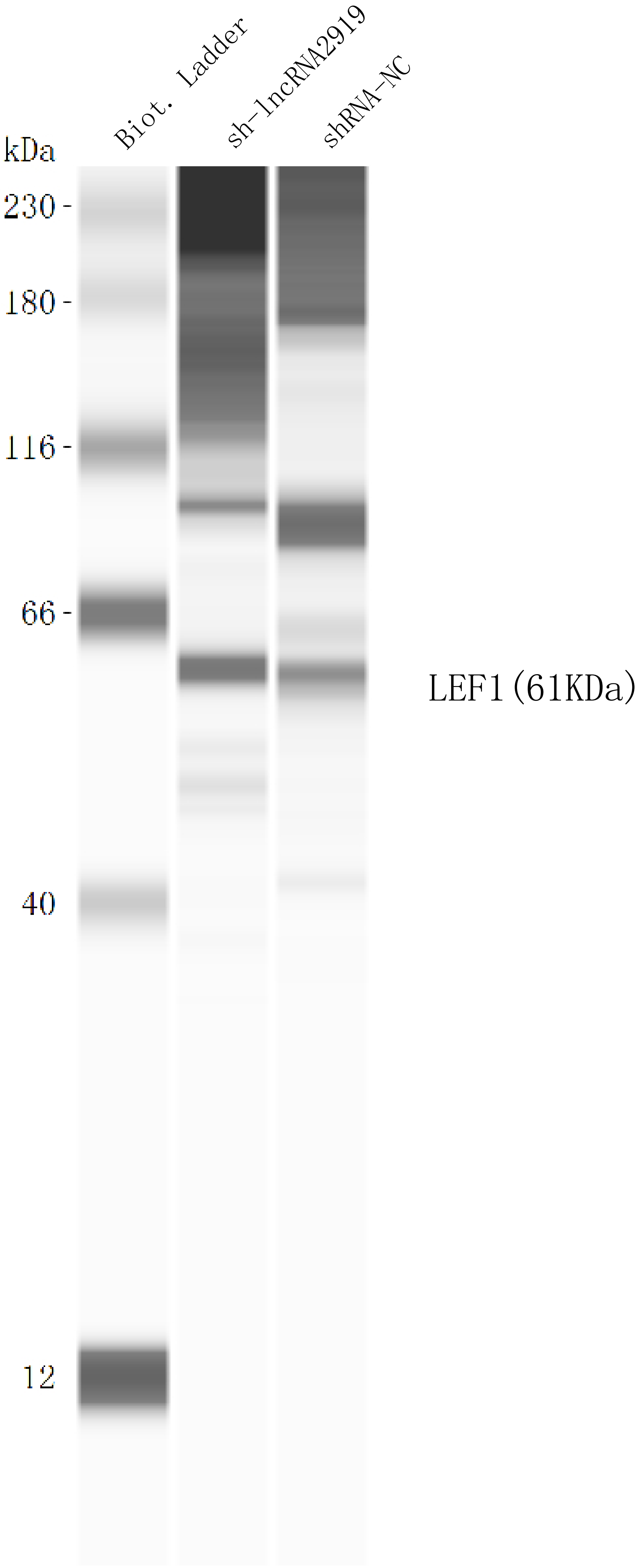

Biot. Ladder
sh-lncRNA2919
shRNA-NC
kDa
230
180
116
66
40
12
LEF1(61KDa)

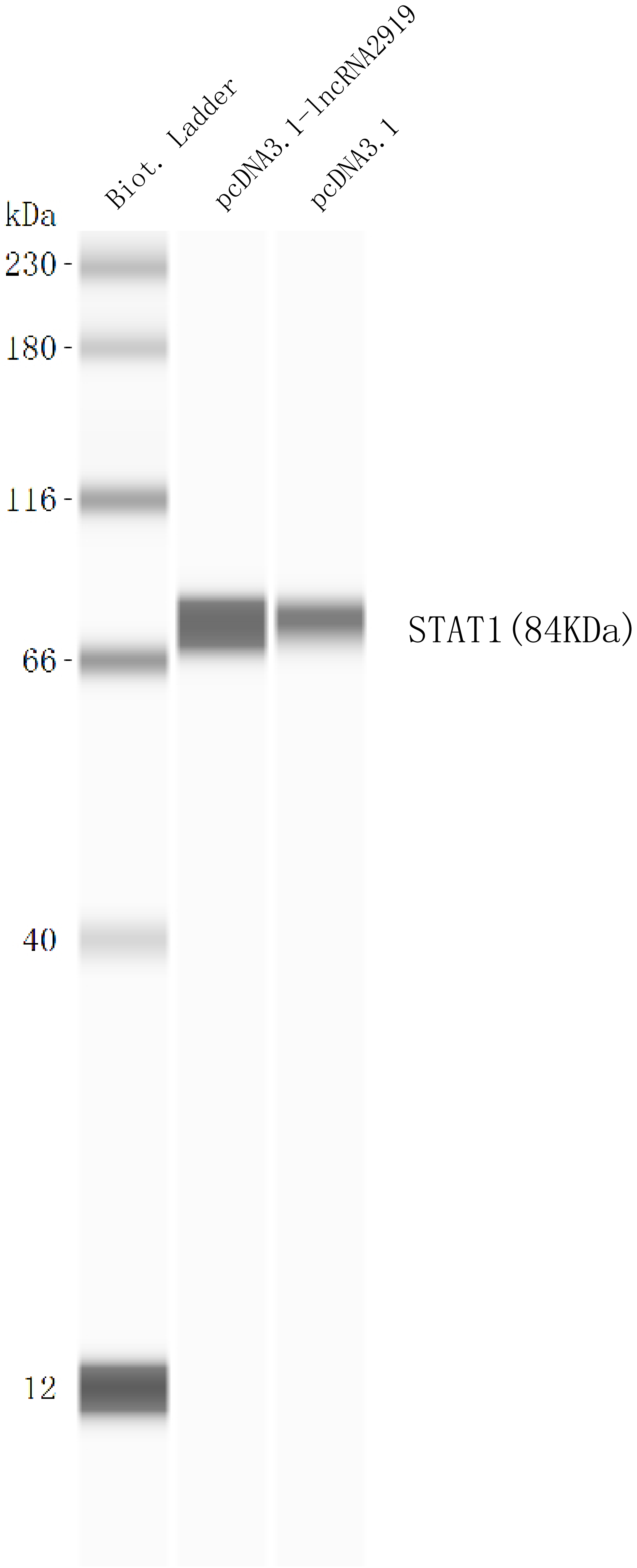
Biot. Ladder
pcDNA3.1-lncRNA2919
pcDNA3.1
kDa
230
180
116
66
40
12
STAT1(84KDa)

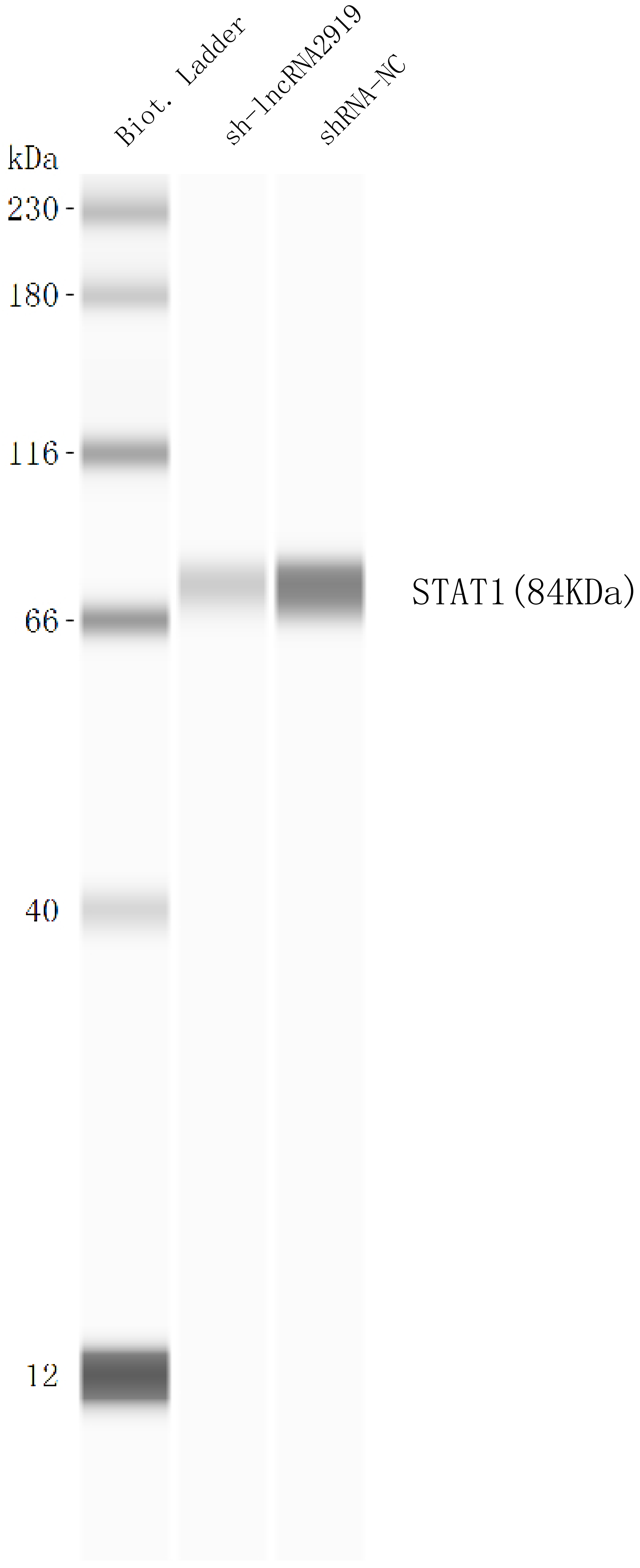

Biot. Ladder
sh-lncRNA2919
shRNA-NC
kDa
230
180
116
66
40
12
STAT1(84KDa)

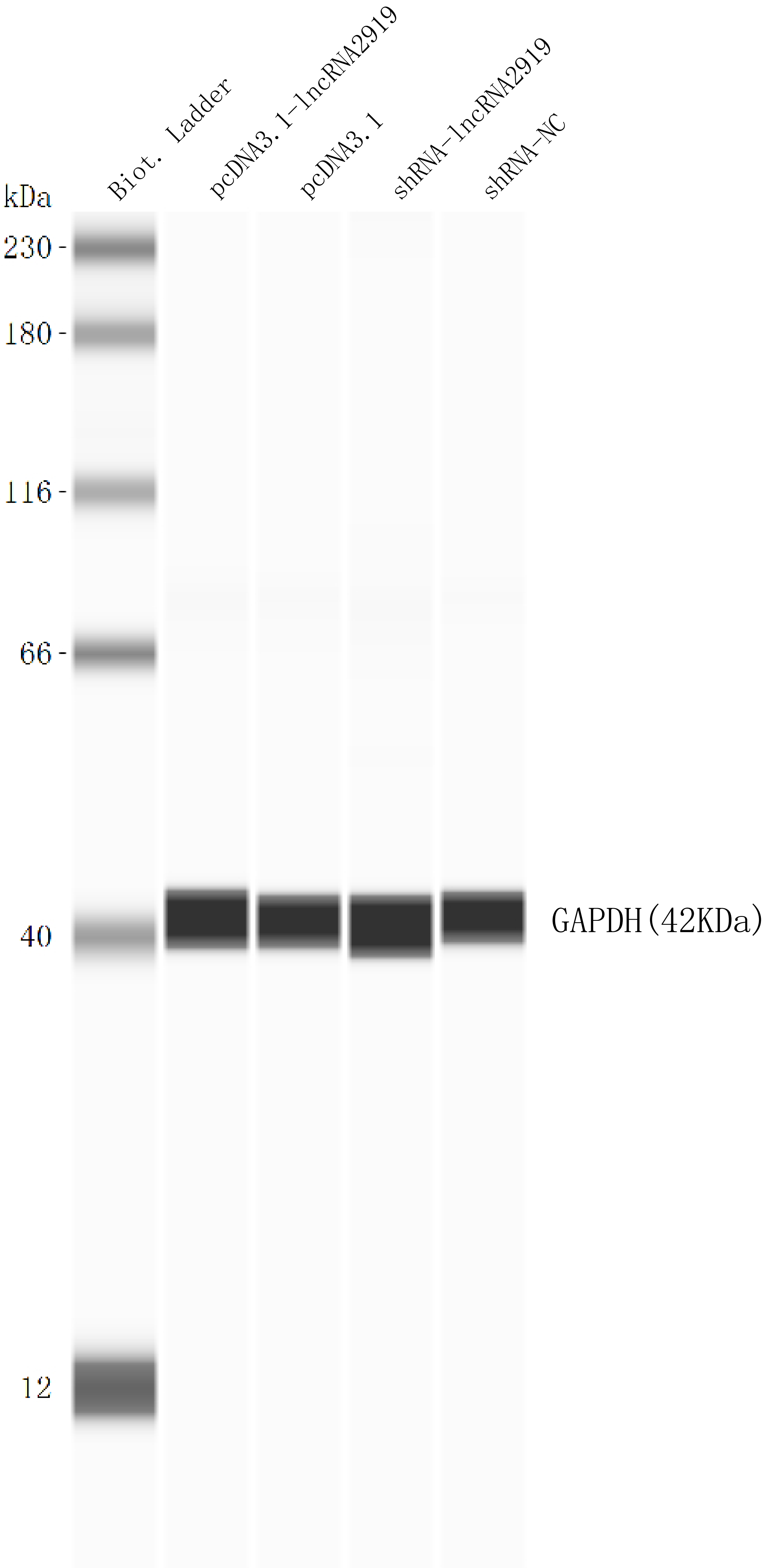

Biot. Ladder
pcDNA3.1-lncRNA2919
pcDNA3.1
shRNA-lncRNA2919
shRNA-NC
kDa
230
180
116
66
40
12
GAPDH(42KDa)

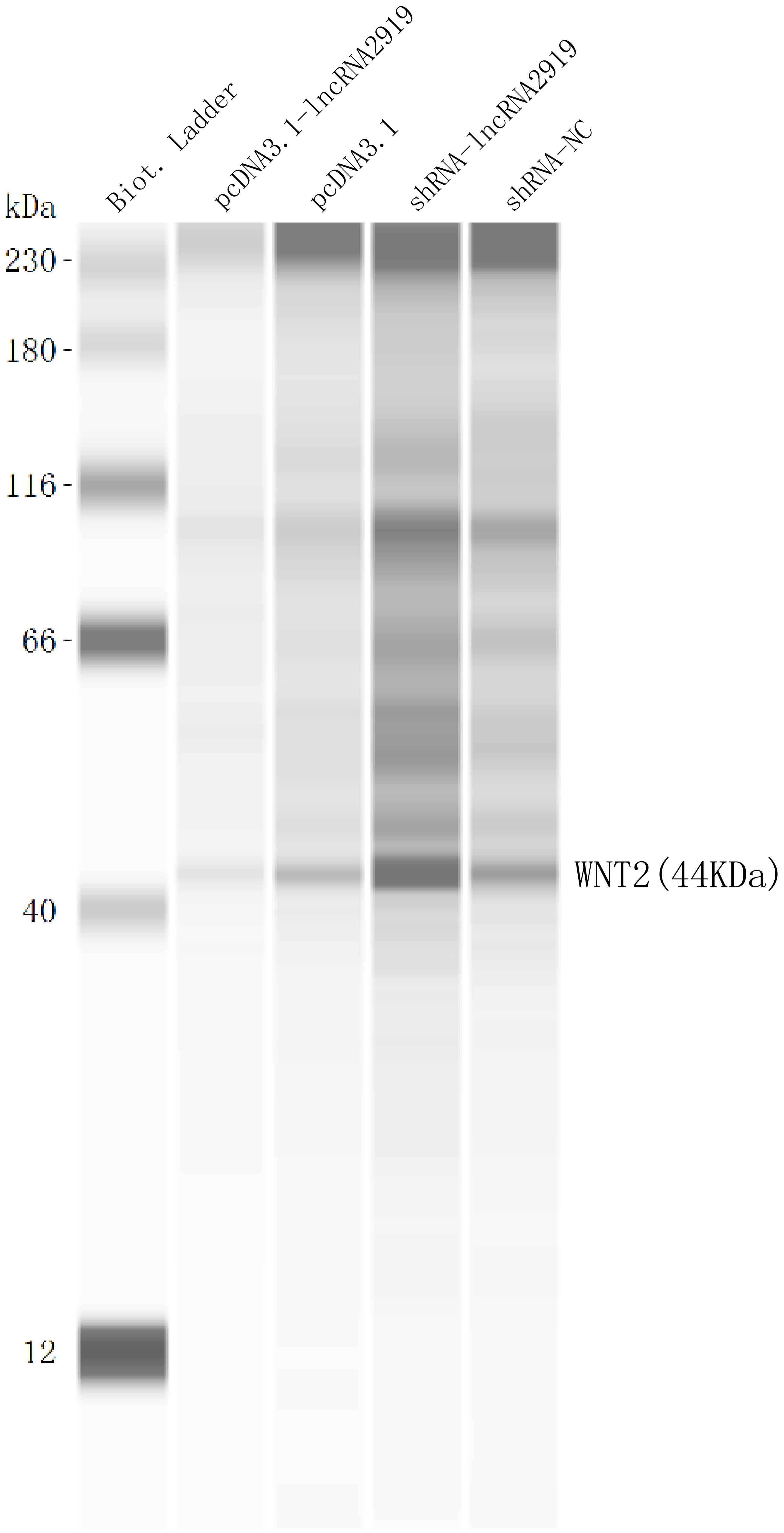
kDa
Biot. Ladder
pcDNA3.1-lncRNA2919
pcDNA3.1
shRNA-lncRNA2919
shRNA-NC
230
180
116
66
40
12
WNT2(44KDa)

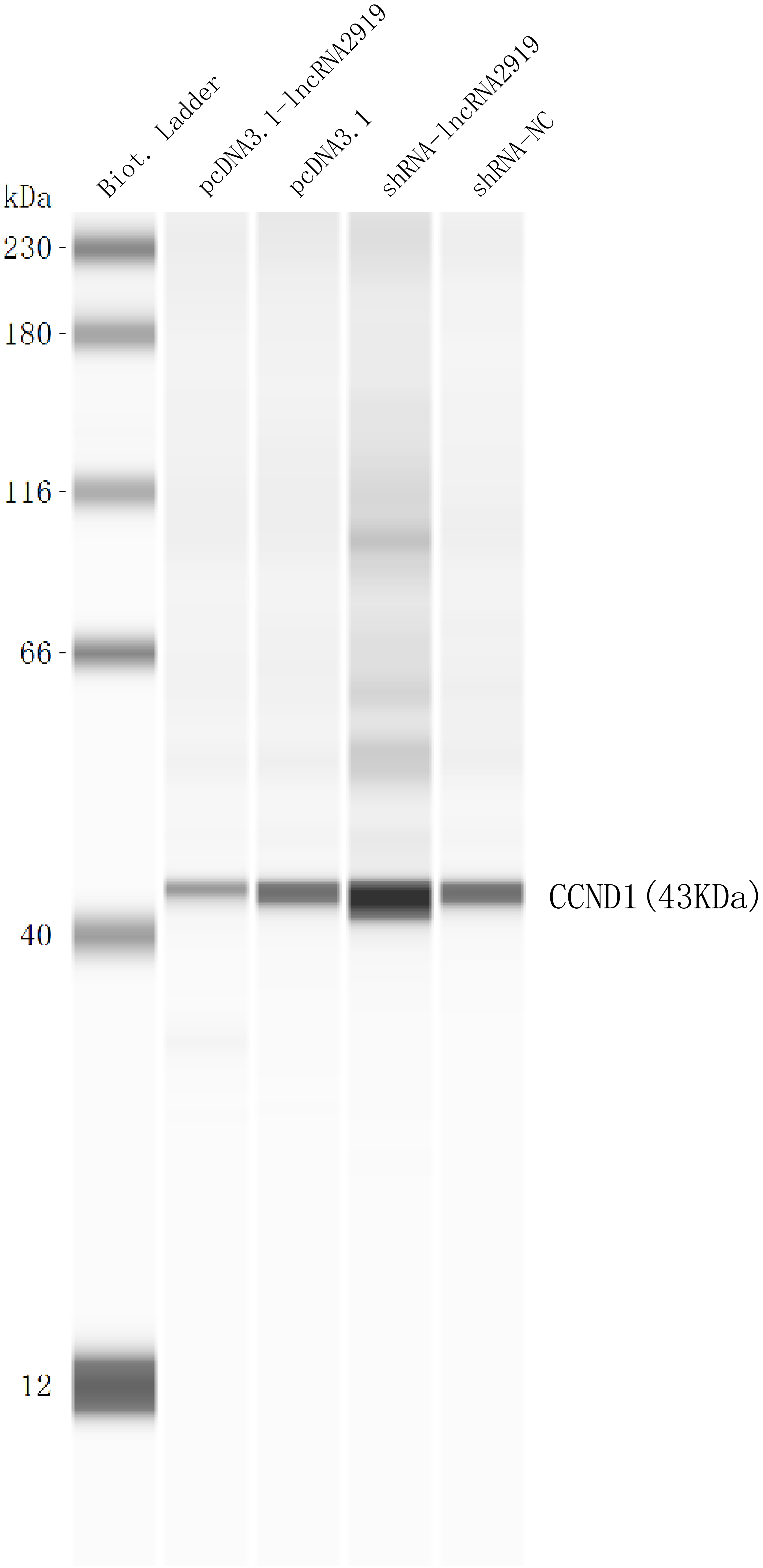

kDa
Biot. Ladder
pcDNA3.1-lncRNA2919
pcDNA3.1
shRNA-lncRNA2919
shRNA-NC
230
180
116
66
40
12
CCND1(43KDa)

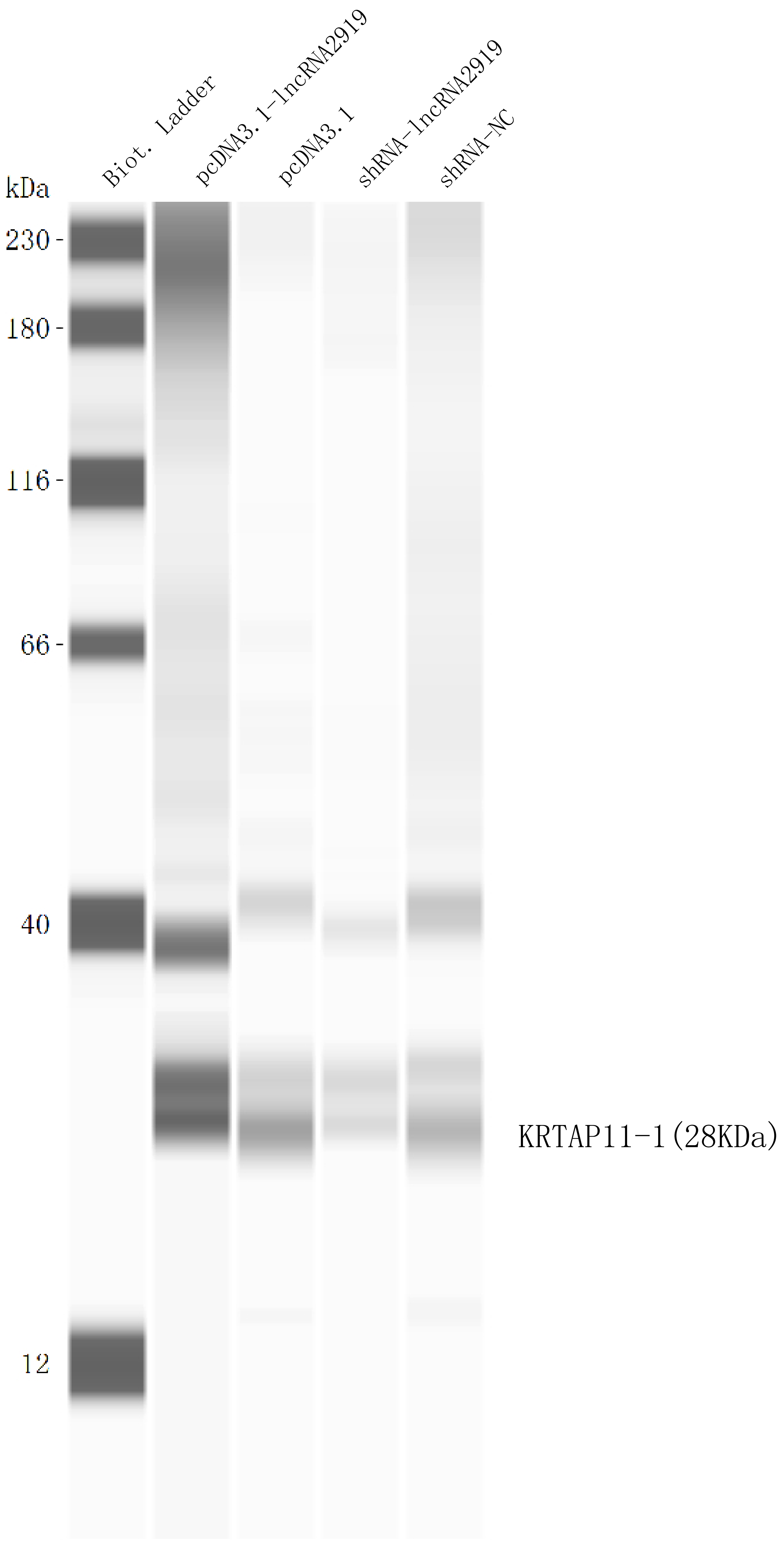
kDa
Biot. Ladder
pcDNA3.1-lncRNA2919
pcDNA3.1
shRNA-lncRNA2919
shRNA-NC
230
180
116
66
40
12
KRTAP11-1(28KDa)

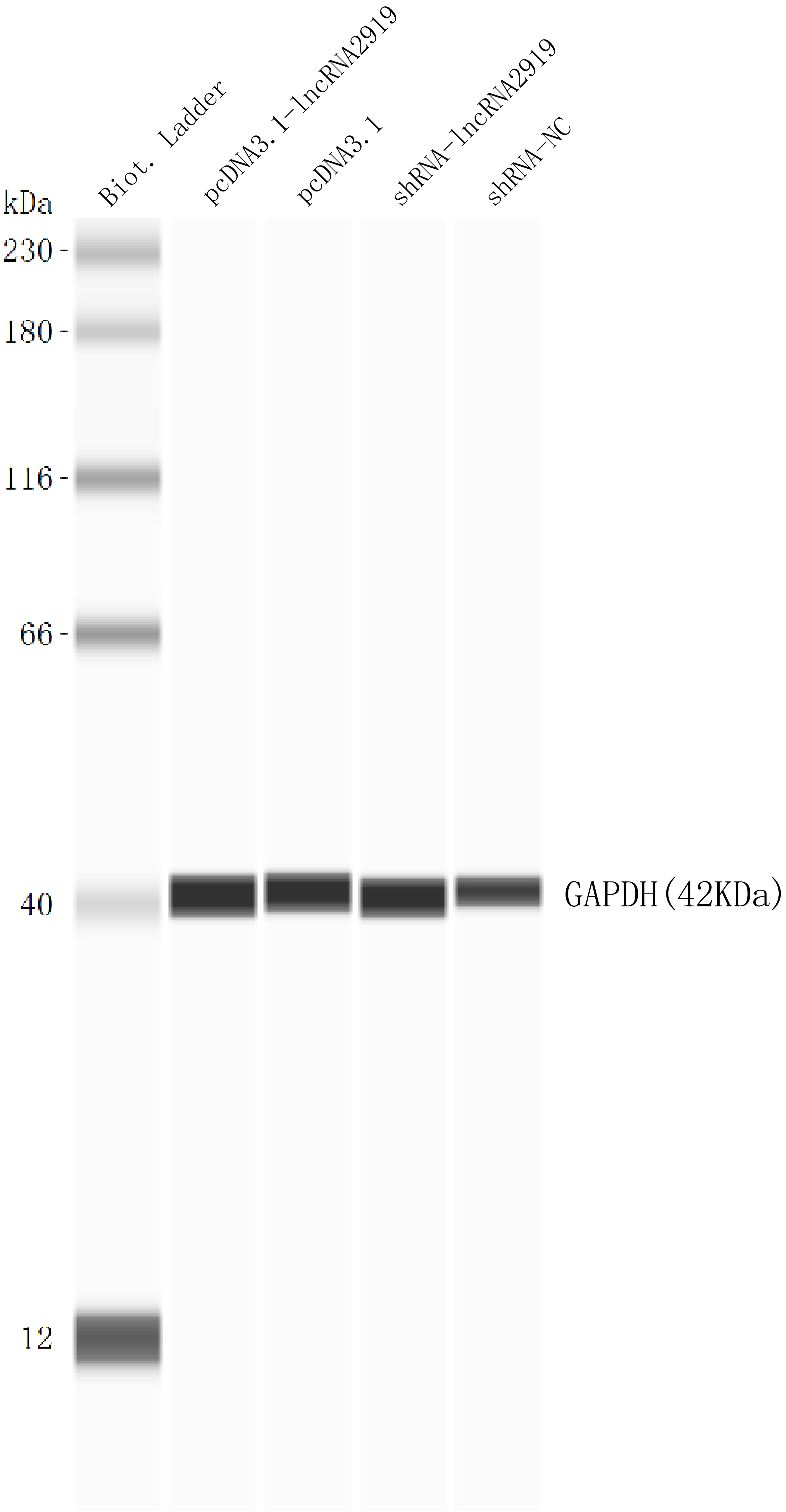
Biot. Ladder
pcDNA3.1-lncRNA2919
pcDNA3.1
shRNA-lncRNA2919
shRNA-NC
kDa
230
180
116
66
40
12
GAPDH(42KDa)

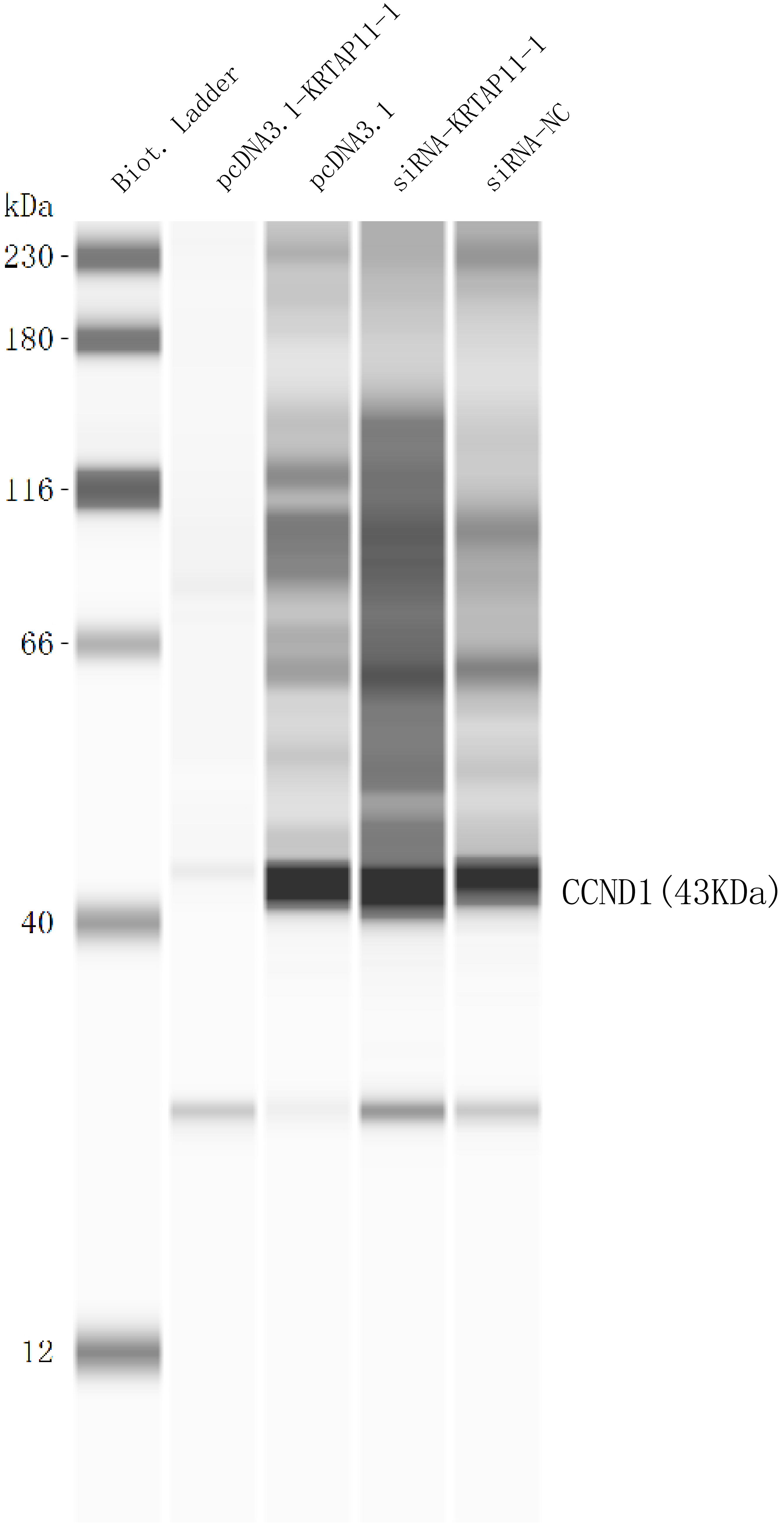
Biot. Ladder
pcDNA3.1-KRTAP11-1
pcDNA3.1
siRNA-KRTAP11-1
siRNA-NC
kDa
230
180
116
66
40
12
CCND1(43KDa)

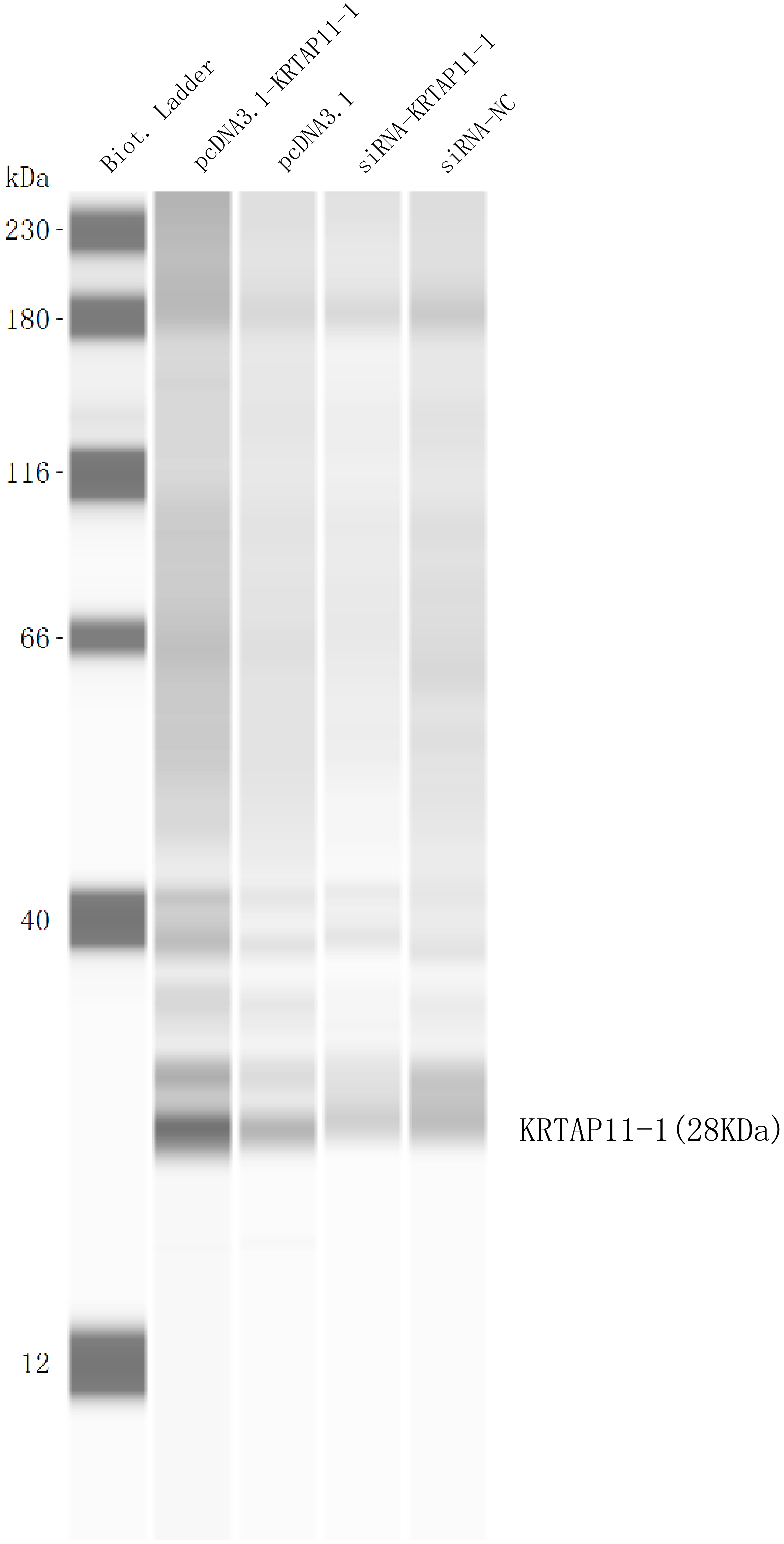
Biot. Ladder
pcDNA3.1-KRTAP11-1
pcDNA3.1
siRNA-KRTAP11-1
siRNA-NC
kDa
230
180
116
66
40
12
KRTAP11-1(28KDa)

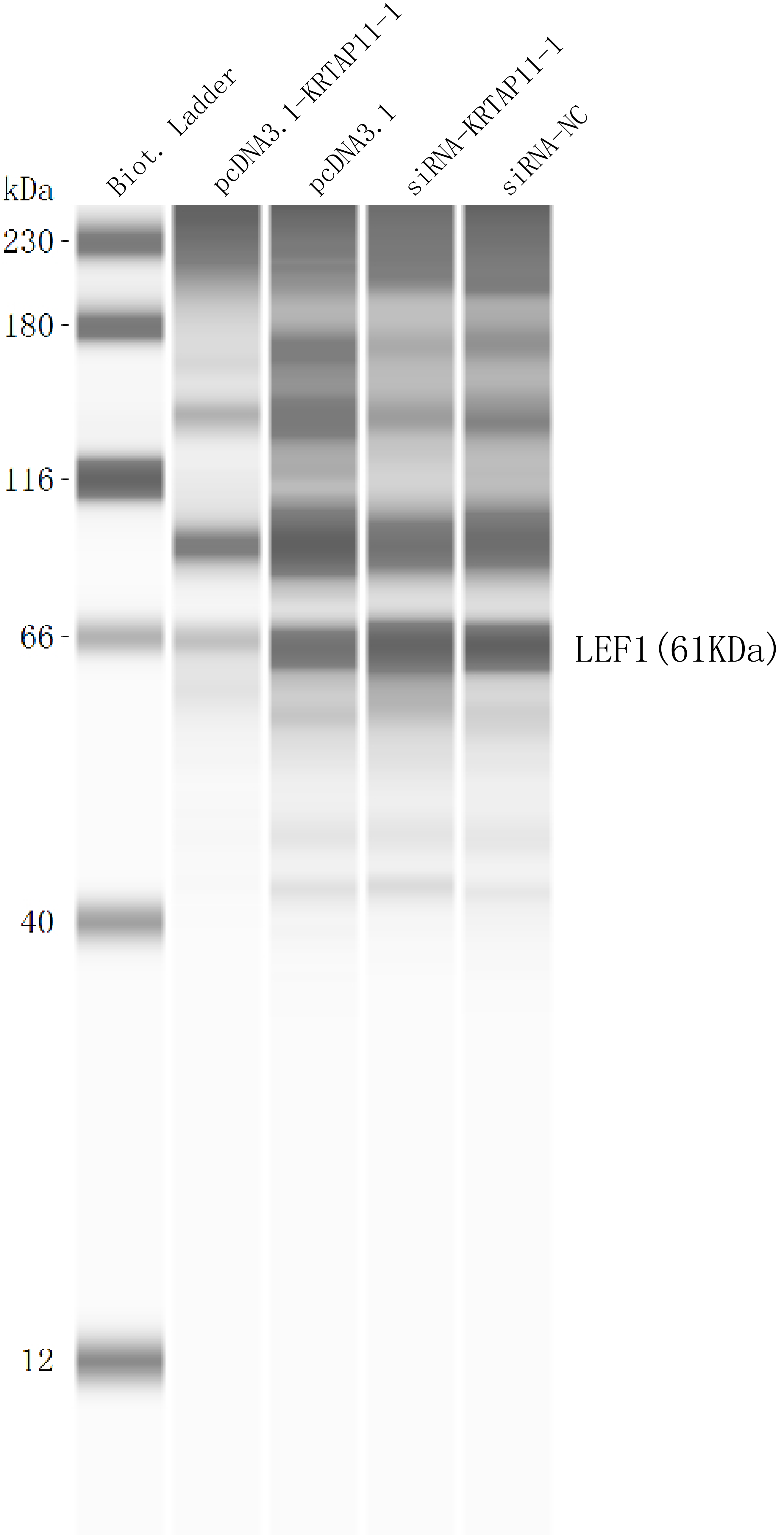
kDa
Biot. Ladder
pcDNA3.1-KRTAP11-1
pcDNA3.1
siRNA-KRTAP11-1
siRNA-NC
230
180
116
66
40
12
LEF1(61KDa)

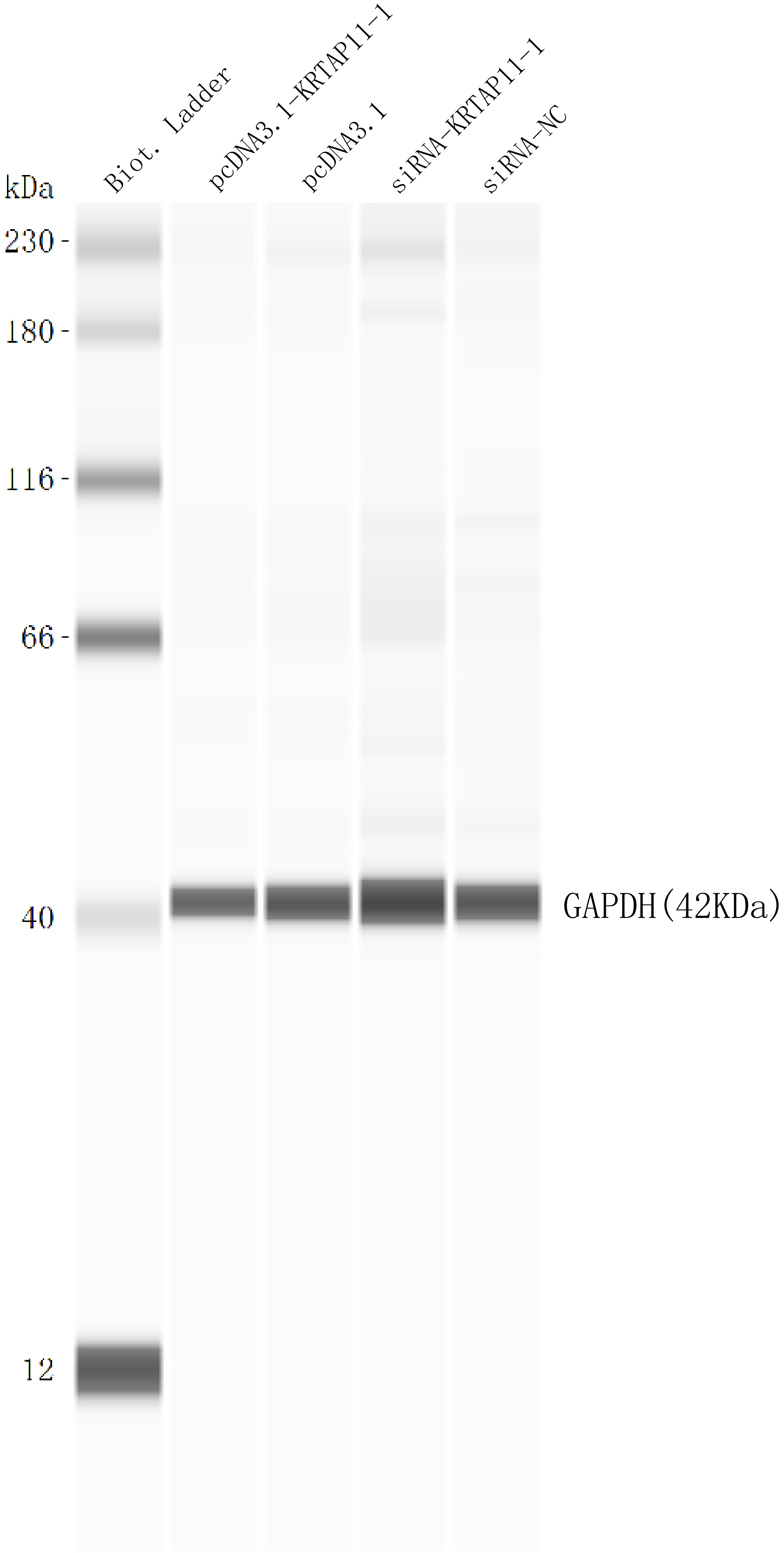
Biot. Ladder
pcDNA3.1-KRTAP11-1
pcDNA3.1
siRNA-KRTAP11-1
siRNA-NC
kDa
230
180
116
66
40
12
GAPDH(42KDa)
