# Supplementary material for: lncRNA2919 Suppresses Rabbit Dermal Papilla Cell Proliferation via trans-Regulatory Actions
Source: Cells. 2022 Aug 6;11(15):2443. doi: 10.3390/cells11152443 (PMC9368379; doi:10.3390/cells11152443)
Supplement: Supplementary file 1 [file cells-11-02443-s001.zip › Supplementary file S2.pdf]

The full-length sequence of lncRNA2919 by 5′/3′ RACE (5’- 3’):

ATACTCAAGAAACTCGTCCTTGACGTCTGACCAAACCTACAGTTCCTCACACCATGTG
CCACCTTGGAAACTACTACGGAGGCCGGAGCTTCGGCTACAGAAGCTTGGGTGGCCT
GGGCTTTGGCCATGGAGGCTTCTGTGGCCTGGGCTTTGGCCATGGAGGTTTCGGTGGC
CTGGGCCTGGGTCATGGCTATGGTTATGGATGTGGCAACTTCCACACATTTGGCTGTG
GCTCTGGGTTTGGAGGCTTTGGACATAGCTACTACCGCCCAGTGTCCTATGGAGGATA
TAGATTCTCTAGGTTCTACTGAAGAGTAAACTTGTTCTTTGGACCATTGGAATCTTCAT
GTCATCGTCATTTAGTTCTGATTCCAGATCCCCGAACTCCCTCTTTCAATTGCACAGCT
GCCTTCTTGATAAACCAGCGAAGGCTGCCTCATGAACTCTTCAAAAGCAAGACTCCAT
TCTGAAAACGAACACCAGAAGGAGCACCTGCAGCAGCAGCAAGAGGAACCCCTCCT
GCAGACTGATCTGGAATCTCATCTTTCAAGCTATCTATCCCTCTCTTCCCCTTTAATAT
CCCAATATGGCACTGACCCCCATGTTTCAATAAATTAATTTCAATAGCACAGCAAATT
TTGTTTCATTTATTTATTTTATTTAAATTTTTTTATTTAAATAAATTATAAAATTTAATT
TATTTTAAACTTTATTTTATTGTGCTAGGATATCAACCCTTAATTTTTGTGTACAGTAC
AGTATTGTTGACTTTAGGAGCAACGCTGTACAGCAGGTCTCTAGAGCTTATTCGTCTT
GCTTGACTGAAACTTTGTGCTGTTTTGTCGGTAACTCTCCATTTCCCTTTCCACTCAGC
CCCAAGTAATCACCATTTCACTCTTTGTCCCCTTGATTTTGACTATTTTCAATACCTCA
TATAAACACCATCCTGTGTTATATTTGTCTTTCTGTGTTTGACATCTTTCACTTGGTATC
ATTACTATTGATTCATTTCCTAGAACCAATCCCAACCTCCCTGATCTGGGGTTGCTCAT
TCATCATCACATGATAGATACCTGGCACACAGAAGGCAGCAGTCAGAGAATATTTGT
GGATTAGGAAATTTGGCTCAAGTCTTTTATCAGAAAGAATAATACATCATCCCAATTT
TGGAATCTTGGCATCCTTTCCTACCTCTTTACCCTCTTCTGGTGTCAATCCTACCCCCA
GCCCCTAACCACACACACACTATCGTTAGACATATCTCGCCCGATATAATTTCTCTTC
ATCTTGGGAATTAACAGAATGCCTCAACAGTAACACTCTTTGTGAAACTCCTTACAGG
ATCAGGAAGAATGCCTTGCAATTTGGCAAAAAAACAGAAGTTGAAAATTATTGACTG
TTGAGGCAAGTTATGGGATGGGGAAAGAAATTTGCAACATATTAAGCCGGCGCCGTG
GCTCACTAGGCTAATCCTCCACCTTGCGGCGCCGGCACACCGGGTTCTAGTCCCGGTT
GGGGCGCCGGATTCTGTCCCGCTTGCCCCTCTTCCAGGCCAGCTCTCTGCTGCGGCCA
GGGAGTGCAGTGGAGGATGGCCCAGGTGCTTGGGCCCTGCACCCCATGGGAGACCAG
GAAAAGCACCTGGATCCTGGCTCCTGCCATCGGATCAGCGCGGTGCGCCGGCTGCAG
CGGCGGCCATTGGAGGGTGAACCAACGGCAAAGGAAGACCTTTCTCTCTCTGTCTCTC
TCTCTCACTGTCCACTCTGCCTGTCAAAAATTAAAAACAAACAAACAAACAAACAAA
AAAAGAAATTTGCAACATATGTAAGTAAAACTGGCAAATGTGCACCCTTGGACAACC
CTGTAAGTAATCCAATGCCTTTCGGCAACAGAATAGAAGCATGAATGTAGTTTGTTCT
TGCAGTGGGGGACGACAAAAAAAAAAAAAAAAAAAAAAAAA

The full-length sequence of KRTAP11-1 mRNA by 5′/3′ RACE (5’- 3’):

TCTCACAACTTAGGAGCTAACTTCACTGAAGTACCTTCAGCCCTCTCTCTGACATCAT
GTCCTTCAGCTGCTCCACAAGAGATTGCTCTTCCAGGCCGATCGGAGGACGGTACACT
GTCCCCGTGGCCCCAGCTGCCACGTCTTCAACCCTTGAAGGCGACTGCCTGAGTAGCA
TCTATTTGCCCAGTTCCTTCCAAACTGGCTCTTGGCTCCTGGACCACTGTCAGGAGTCC
AGCTGCGAGCCTACTGTCTGCCAGCCTACTTGTTTCCAGCGAACTTCTTGCATCTCCA
GCCCTGCGCAGGTAACTTGCTCGCGGCAAACTACCTGTGTCTCCAATCCCTGCTCAAC
TGCCAGCAGCCGACCATTCAGCTTTGTCTCAAGTGGCTGTCAGCCCGTGGGAGGCATC

TCCAGTGTCTGCCAGCCAGTGGGCAGCATCTCGACTGTCTGCCAACCAGTGGGAGGA
GTCTCTTCTGTCTGCCAACCAACCTGTGGGTACTCCCGGACGTACCAGCAGTCCTCCT
GTGTGTCCAGCTGCCGAAGAACTTGCTAAGTGTGTAGGATCCAGTGAGTCAAGACTC
CGTGACCTGCCAGCTGTGCTTCCAGGATTGTCCAGCATGCTGCCTGAAAACTCTTCAT
TGCTAAACACTATACTGACTGCCTGATTGCTGGCTGCTAGCCATGCACAAGCCGCCTT
TGGCATGCTCAAAATTTTCTAGCCAGCACCAAGCTTATTTTAAGGGTTGGCCACTGGT
GCTATGCAGTCGCTGGATGTTTCCAGAAGCTTTTTCATTCATGCCACAGGCTCTGATA
ATTTTGTCATGTTTTGACCTTGCTGCTGAATCTCCTGACCTCTGCTTCTGTGTCTGTGA
AAGAGGGAGCTTGTCTCACTATGTATTTCTCAATAAACCTGCATTAGTTGGCATTGCA
AAAAAAAAAAAAAAAAAAAA

The CDS sequence of STAT1 mRNA by cDNA cloning (5’- 3’)：
ATGTCTCAGTGGTATGAGCTTCAGCAGCTCGACTCTAAATTCCTGGAGCAGGTTCACC
AGCTCTATGATGACAGTTTTCCCATGGAAATTCGACAGTACCTGGCACAGTGGCTAGA
AAATCAGGACTGGGAGCACGCCGCCAATGATGTTTCGTTTGCCACCATCCTCTTCCAT
GACCTGCTCTCGCAGCTGGACGATCAGTACAGTCGCTTCTCTCTGGAGAACAATTTTC
TGTTGCAGCATAACATAAGGAAAAGCAAGCGGAACCTTCAGGACAATTTTCAAGAAG
ACCCCATACAGATGTCTATGATCATCTATAACTGTCTGAAGGAGGAGCGGAAGATTC
TGGAAAATGCGCAGAGATTCAATCAGGCCCAGTCGGGGAATATTCAGAGCACCGTGA
TGTTAGACAAGCAGAAGGAGCTTGACAACAAAGTCAGAAATGTGAAGGAGCAAGTG
ATGAATATAGAGCACGAAATCAAGACCCTGGAAGATCTACAAGATGAGTATGACTTC
AAGTGCAAAACCTTGCAGAACCGAGAACATGAAGCCAATGGTGTGGCAAAGAGCGA
CCAGAAGCAAGAGCAGCTGCTGCTCCAGAAGATGTATTTAATGCTGGACAACAAGAG
GAAGGAAATCGTTCACAAGATGAGAGAGTTGTTGAATGCCACTGAGCTCACCCAGAA
TGCCCTGATTAACGATGAGCTCGTGGAGTGGAAGCGGAGGCAGCAGAGCGCCTGTAT
CGGGGGCCCGCCCAACGCGTGCCTGGACCAGCTGCAGAACTGGTTCACCATAGTGGC
AGAGAGTCTGCAGCAGGTTCGTCAGCAGCTTAAAAAGCTGGAGGAATTGGAACAGA
AATTCACCTATGAACATGACCCTATCACCAAGAACAAACAGGTGCTATGGGATCGCA
CCTTCAGTCTCTTCCAGCAGCTCATTCAGAGCTCGTTCGTGGTGGAGAGACAGCCTTG
CATGCCCACTCATCCTCAGAGACCGCTGGTCTTGAAGACTGGGGTGCAGTTCACTGTG
AAGCTGAGGCTGTTGGTGAAACTGCAAGAGCTGAATTATAACTTGAAAGTCAAAGTC
CTGTTTGACAAAGACGTGAATGAGAGAAACACAGTGAAAGGATTCAGGAAGTTCAAC
ATCCTGGGCACACACACGAAGGTGATGAACATGGAGGAGTCCACCAACGGAAGTCTG
GCGGCGGAATTCCGACACCTGCAACTGAAAGAACAGAAGAACGCTGGCAACAGGAC
TAACGAGGGTCCTCTAATTGTAACCGAAGAGCTTCACTCCCTAAGTTTTGAAACCCAG
CTGTGCCAGCCTGGCCTGGTCATTGACCTCGAGACGACTTCCCTGCCCCTCGTGGTGA
TCTCCAACGTCAGCCAGCTCCCGAGCGGTTGGGCGTCCATCCTGTGGTACAACATGCT
GGTGGCGGAGCCTAGGAATCTGTCCTTCTTCCTGAACCCACCGTGTGCGCGATGGTCT
CAGCTCTCGGAGGTACTGAGTTGGCAGTTTTCCTCTGTCACCAAACGCGGCCTCAACG
GGGACCAGCTGAGCATGCTGGGAGAGAAGCTACTTGGTCCTAATGCCGGCCTCGATG
GTCTTATTCCGTGGACGAGGTTCTGCAAGGAAAATATAAGTGACAAAAATTTCTCCTT
CTGGCTGTGGATTGAAAACATCCTAGAACTGATTAAAAAGCACCTGCTGTGTCTATGG
AATGATGGGTGCATCATGGGCTTCATCAGCAAGGAGCGAGAGCGCGCCCTGCTGAAG
GACCAGCAGCCCGGCACGTTCCTGCTGCGGTTCAGCGAGAGCAGCCGGGAAGGCGCC

ATCACGTTCACGTGGGTGGAGCGGTCCCAGAACGGAGGCGAACCTTATTTCCATGCG
GTTGAGCCCTACACGAAGAAAGAACTTTCTGCTGTGACTTTCCCTGATATCATCCGCA
ATTACAAAGTCATGGCTGCCGAGAATATTCCTGAGAACCCCCTGAAGTATCTGTATCC
AAATATTGACAAGGACCATGCCTTCGGGAAGTATTACTCCAGGCCAAAGGAAGGGCA
AGAACCCATGGAACTTGACGGCCCCAAAGGAACTGGATACATCAAGACTGAGTTGAT
TTCTGTGTCTGAAGTTCACCCTTCTAGACTCCAGAGCACAGAAAACCTGCTGCCCATG
TCTCCTGAGGAGTTTGATGAGGTGTCTCGGATAATGGGCCCTGTAGAATTCGACAGTA
TGATGAACGCAGTATAG
