# Supplementary figures and images for: lncRNA2919 Suppresses Rabbit Dermal Papilla Cell Proliferation via trans-Regulatory Actions
Source: Cells. 2022 Aug 6;11(15):2443. doi: 10.3390/cells11152443 (PMC9368379; doi:10.3390/cells11152443)

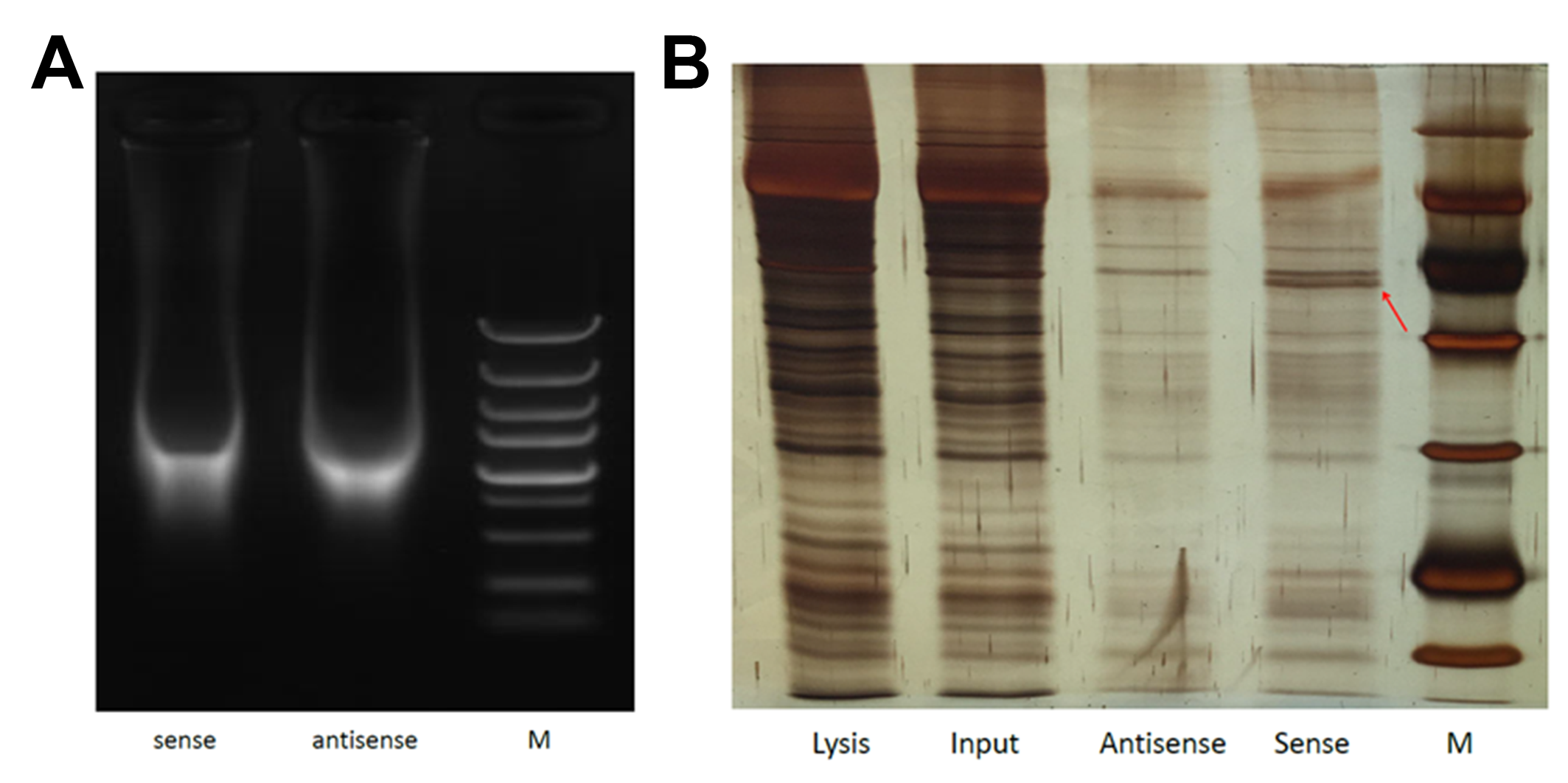

Supplement: Supplementary file 1 [file cells-11-02443-s001.zip › Figure S1.tif]

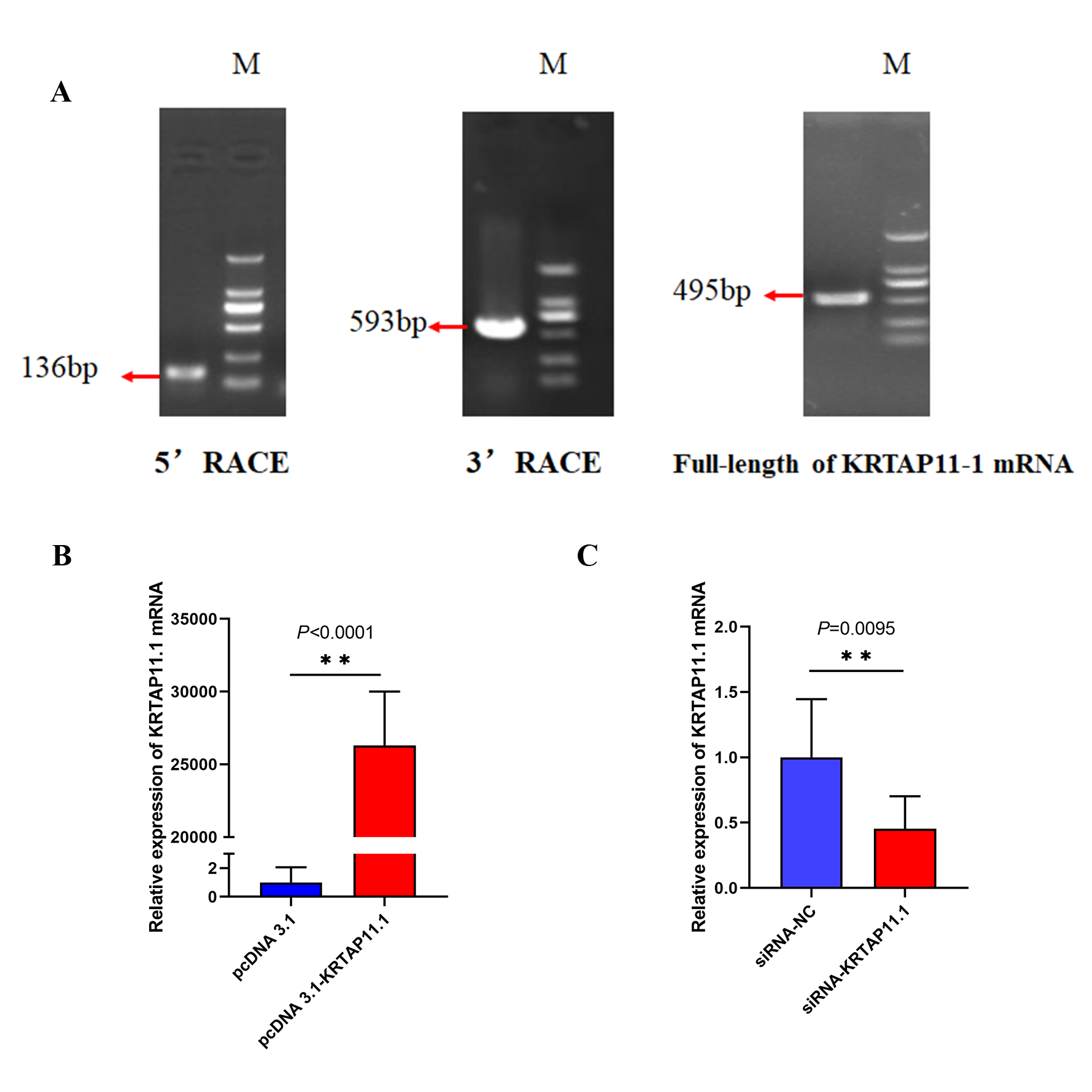

Supplement: Supplementary file 1 [file cells-11-02443-s001.zip › Figure S2.tif]

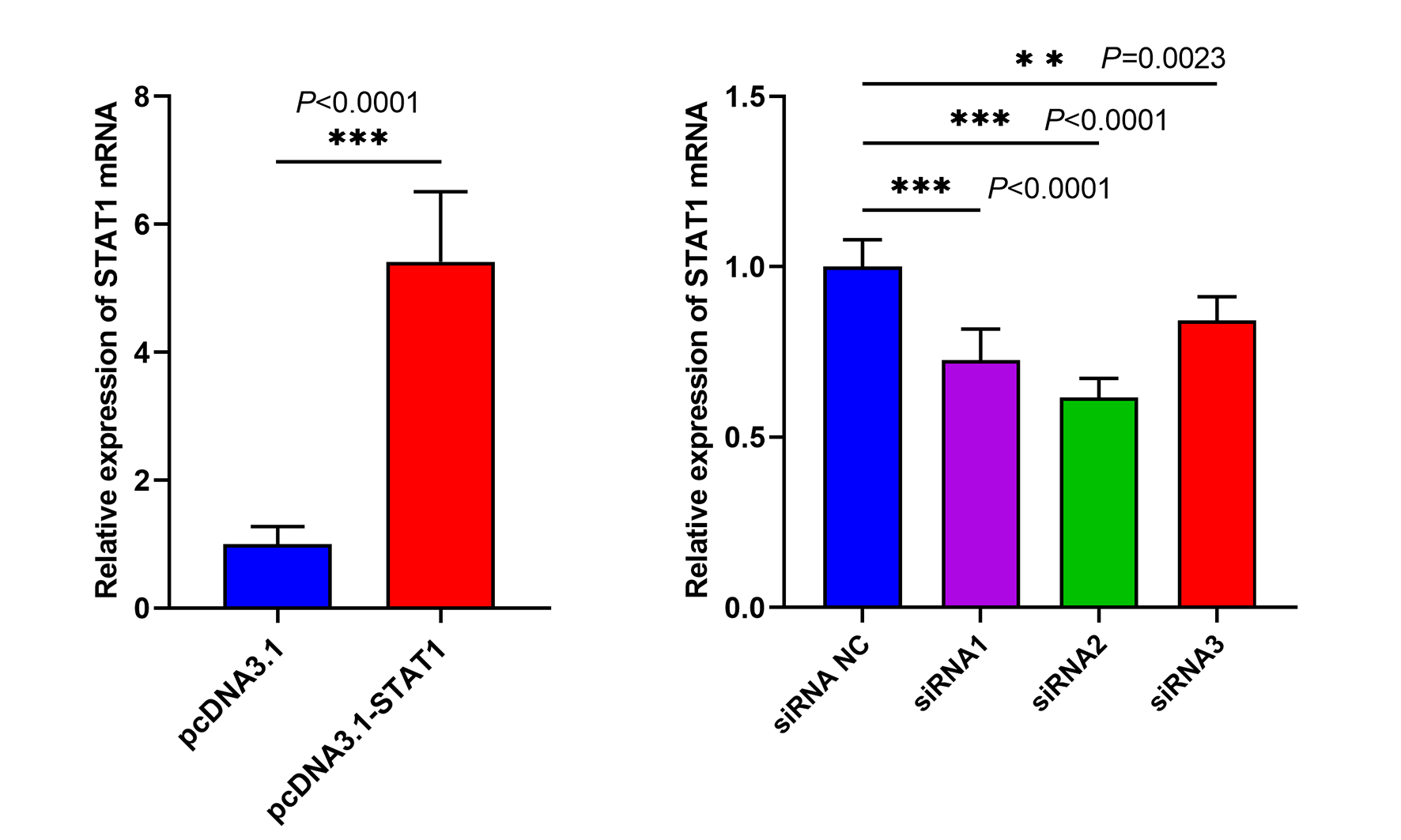

Supplement: Supplementary file 1 [file cells-11-02443-s001.zip › Figure S3.tif]
